# Integrating probabilistic models of perception and interactive neural networks: a historical and tutorial review

**DOI:** 10.3389/fpsyg.2013.00503

**Published:** 2013-08-20

**Authors:** James L. McClelland

**Affiliations:** Department of Psychology and Center for Mind, Brain, and Computation, Stanford UniversityStanford, CA, USA

**Keywords:** interactive activation, context in perception, neural networks, probabilistic computation, generative models

## Abstract

This article seeks to establish a rapprochement between explicitly Bayesian models of contextual effects in perception and neural network models of such effects, particularly the connectionist interactive activation (IA) model of perception. The article is in part an historical review and in part a tutorial, reviewing the probabilistic Bayesian approach to understanding perception and how it may be shaped by context, and also reviewing ideas about how such probabilistic computations may be carried out in neural networks, focusing on the role of context in interactive neural networks, in which both bottom-up and top-down signals affect the interpretation of sensory inputs. It is pointed out that connectionist units that use the logistic or softmax activation functions can exactly compute Bayesian posterior probabilities when the bias terms and connection weights affecting such units are set to the logarithms of appropriate probabilistic quantities. Bayesian concepts such the prior, likelihood, (joint and marginal) posterior, probability matching and maximizing, and calculating vs. sampling from the posterior are all reviewed and linked to neural network computations. Probabilistic and neural network models are explicitly linked to the concept of a probabilistic generative model that describes the relationship between the underlying target of perception (e.g., the word intended by a speaker or other source of sensory stimuli) and the sensory input that reaches the perceiver for use in inferring the underlying target. It is shown how a new version of the IA model called the multinomial interactive activation (MIA) model can sample correctly from the joint posterior of a proposed generative model for perception of letters in words, indicating that interactive processing is fully consistent with principled probabilistic computation. Ways in which these computations might be realized in real neural systems are also considered.

## Introduction

For well over a century (Huey, 1908), there has been an interest in understanding how context affects the perception of the spoken and written word. During the cognitive revolution of the 1950's and 60's, George Miller and others contributed important findings (e.g., Miller et al., 1951; Tulving et al., 1964) showing that context facilitated word recognition, and these findings were captured in the classical Logogen model (Morton, 1969). Reicher (1969) introduced the striking word superiority effect, demonstrating that letters are perceived more accurately in words than in isolation, and the phenomenon received extensive investigation in the early 1970's (e.g., Wheeler, 1970; Aderman and Smith, 1971; Johnston and McClelland, 1973, 1974; Thompson and Massaro, 1973). Rumelhart and Siple (1974) and Massaro (1979) offered models of context effects in letter perception, and Rumelhart (1977) laid out how such a model might be extended to address a broader range of contextual effects, including syntactic and semantic effects and effects of non-linguistic context on word identification and semantic interpretation.

The models mentioned above were all either explicitly probabilistic models or could be linked easily with probabilistic, Bayesian computations. But then a funny thing happened. On the one hand, Pearl (1982) offered a systematic Baysian framework that unified the earlier models into an general algorithm (subject to some limitations) for probabilistic Bayesian inference across multiple mutually interdependent levels of interpretation (feature, letter, word, syntactic/semantic interpretation). On the other hand, Rumelhart and I diverged from the path of probabilistic Bayesian models, proposing a model of context effects in letter perception (McClelland and Rumelhart, 1981) that did not refer explicitly to probabilistic Bayesian ideas, drawing inspiration, instead, from models of neural activation (Grossberg, 1978). In fact, as Massaro (1989) pointed out, our interactive activation (IA) model actually failed to account for aspects of data that were easily captured by the earlier models and by simple Bayesian considerations.

A considerable debate ensued, one in which it seemed for a while as though there might be an intrinsic conflict between probabilistic Bayesian models on the one hand and not just connectionist models but *any* model involving bi-directional propagation of influences on the other. Pearl's work clearly provided an interactive method of carrying out provably valid probabilistic Bayesian computations, but Massaro (1989); Massaro and Cohen (1991) as well as Norris and co-authors (Norris et al., 2000) nevertheless argued that bi-directional propagation of information would lead to violations of correct probabilistic Bayesian inference. While I and my collaborators (McClelland, 1991; Movellan and McClelland, 2001; McClelland et al., 2006) were able to address many of the specific criticisms, the notion that distortion of valid inference is intrinsic to bi-directional propagation of information has persisted (Norris and McQueen, 2008).

In part, this debate reflects a simple failure on the part of psychologists (including myself!) to keep up with developments in computer science and related disciplines, and in part, it reflects an enthusiasm represented by early neural network models to draw inspiration from putative principles of brain function rather than principles of probabilistic inference. In any case, the purpose of the current article to establish a reconcilliation. Specifically, I seek to reassure those who stand firm for principled Bayesian models and those who seek inspiration from principles of brain-like processing that both sides can be happy at the same time.

The path I will take toward furthering this rapprochement will begin by introducing basic principles of probabilistic Bayesian inference and then indicating how these principles can be instantiated in models that also adopt principles of brain-like processing. The presentation is in part tutorial and in part historical, and is intended to help put experimentally oriented cognitive scientists, neural network modelers, and proponents of probabilistic Bayesian computation on the same page with respect to the relationship between models of perception, neural networks, and Bayesian inference.

Many of the concepts that will be reviewed are instantiated in a new version of the IA model of letter perception (McClelland and Rumelhart, 1981) called the multinomial interactive activation (MIA) model (Khaitan and McClelland, 2010; Mirman et al., in press), and that model will be used as a vehicle for discussion of these issues. The MIA model (like the IA model before it) can be viewed as a simplified model of the process of inferring the identities of objects in the external world (in this case, words and the letters of which these words are composed) from noisy visual input, and models based on the IA model and related interactive activation and competition networks (McClelland, 1981) are widespread in psychological research on topics ranging from written and spoken word perception (Elman and McClelland, 1988; Grainger and Jacobs, 1996), face perception (Burton et al., 1990), and memory retrieval (Kumaran and McClelland, 2012) to construal of personality (Freeman and Ambady, 2011). The development here will connect the intuitive principles of contextual influences on perceptual identification that were embodied in the original IA model with Bayesian ideas, showing how the new variant of the original model (the MIA model) provides a system for principled probabilistic inference similar to that envisioned in a precursor to the IA model by Rumelhart (1977) and systematized by Pearl (1982). The ideas draw heavily on the original framing of the Boltzmann Machine (Hinton and Sejnowski, 1983). They are related to ideas presented by Lee and Mumford (2003) and Dean (2005) that point out connections between Bayesian computational frameworks and real neural networks in the brain, and share several of the ideas underlying deep belief networks (Hinton and Salakhutdinov, 2006), which are, similarly, models of perceptual inference.

Taken together, the ideas we will develop provide a bridge between neurophysiological ideas and cognitive theories, and between probabilistic models of cognition and process-oriented connectionist or parallel-distributed processing models. Thus, this tutorial may prove useful as an introduction for those interested in understanding more about the relationship between a simple form of Bayesian computation and both real and artificial neural networks. While the specific examples are all drawn from perception of letters in words, the possible applications include many other perceptual problems as well as the more general problem of inferring underlying causes from observed evidence.

We begin by presenting Bayes' formula as a tool for inferring the posterior probability that some hypothesis is true, given prior knowledge of certain probabilistic quantities and some evidence^1^. This part of the presentation starts with the case of two mutually exclusive and exhaustive hypotheses and a single source of evidence, and shows how Bayes' formula follows from the definition of conditional probability. We then extend the formula to cover cases involving an arbitrary number of mutually exclusive and exhaustive hypotheses and to cases involving more than one element of evidence, introducing the concept of conditional independence. We then develop the idea of a generative model within which the quantities needed to infer posterior probabilities can be seen as representing parameters of a causal process that generates the inputs to a perceptual system.

We next consider how Bayesian inference can be carried out by a neural network. In particular, we observe how the softmax and logistic activation functions often used in neural networks can produce outputs corresponding to posterior probabilities, provided that the biases and connection weights used in producing these outputs represent the logarithms of appropriate probabilistic quantities.

With the above background, we then describe how bottom-up and top-down information can be combined in computing posterior probabilities of letters presented in context, in accordance with Bayes' formula and the generative model assumed to underlie the perceptual inputs to the MIA model. We describe three procedures by which such posteriors (or samples from them) can be computed—one that is completely non-interactive [appearing to accord with the proposals of Massaro (1989) and elsewhere, and of Norris and McQueen (2008)], and two that involve bi-directional propagation of information, as in the original IA model (McClelland and Rumelhart, 1981). One of these procedures computes these posteriors exactly, and relates to proposals in Rumelhart (1977) and Pearl (1982). The other samples from the posterior, using Gibbs sampling as in the Boltzmann machine (Hinton and Sejnowski, 1983); this is the approach taken in the MIA model. The connection to deep belief networks is considered briefly at the end of the article.

As can be seen from the citations above, the key ideas reviewed here have been in circulation for about 30 years. These ideas establish an intimate connection between the computations performed by neural networks and computations necessary to carry out correct probabilistic inference. Unfortunately, to my knowledge there has not been extensive recognition of these connections, at last among many researchers working in the psychological and cognitive science disciplines. The presentation draws on an earlier paper with similar goals (McClelland, 1998) and is intended to help provide an intuitive understanding of some of the relevant concepts involved, and of the reasons why certain things are true, without relying on formal proofs.

## Using bayes' formula to infer posterior probabilities

We begin by reviewing the canonical version of Bayes' formula, expressing the posterior probability that one of two mutually exclusive and exhaustive hypotheses is true given some evidence *e* in terms of other quantities which we will shortly define:

(1) $$\begin{array}{l} p(h_{i}|e)=\frac{p(h_{i})p(e|h_{i})}{p(h_{1})p(e|h_{1})+p(h_{2})p(e|h_{2})} \end{array}$$

In this expression, *p*(*h*_*i*_) corresponds to the prior probability that hypothesis *i* is true, where *h*_*i*_ could be hypothesis 1 or hypothesis 2. *p*(*e*|*h*_*i*_) corresponds to the probability of the evidence given that hypothesis *i* is true, and *p*(*h*_*i*_|*e*) corresponds to the posterior probability of hypothesis *i* given the evidence. The expression is often called “Bayes' law,” or “Bayes' rule,” although some use “Bayes' rule” for a formulation that expresses the ratio of the posterior probability of *h*_1_ to *h*_2_. Bayes' rule in that form is easily derived from Bayes' formula and *vice versa*. The formula is also sometimes described as “Bayes' Theorem,” but we will use that phrase to refer to the proof of the validity of the formula, rather than the formula itself.

As an example [from the Wikipedia entry on (Bayes' theorem, n.d.)], suppose a friend of yours meets a person with long hair. What is the probability that this person is a woman? Our two possible hypotheses here are that the person is a woman or that the person is a man. We treat them as mutually exclusive and exhaustive—that is, a person must be either a man or a woman; there are no other possibilities, and the person cannot be both a man and a woman at the same time. The evidence *e* is that the person has long hair.

Bayes' formula allows us to calculate the answer to this question, as long as some additional relevant facts are known. First, we need to know the overall probability that a person your friend might meet is a woman. We could call this probability *p*(*h*_1_), but to aid maintaining contact with the example, we will call it *p*(*W*). Since we have assumed that the only other possibility is that the person is a man, the probability that the person is not a woman $p(\bar{W})$ is equal to the probability that the person is a man, *p*(*M*). From this it follows that *p*(*W*) + *p*(*M*) = 1, and that $p(M)=p(\bar{W})=1-p(W)$.

The quantity *p*(*W*) represents to a given or assumed quantity corresponding to the overall probability that a person your friend might meet is a woman. This quantity is often called the *prior*, a usage that makes sense if our goal is to use evidence to update our beliefs about the probability that a person your friend might meet is a woman once we observe the particular person's gender. Here, we are just using this quantity as a premise in an inference process. Nevertheless, writers often use the term prior when describing such terms, and we will often do so here. Another phrase that is sometimes used is *base rate*. Humans often neglect base rates in carrying out probabilistic inference when given probabilistic information in explicit form. When the base rate is low, this can lead to an over-estimate of the posterior probability.

It might be noted that there could be uncertainty about the prior or base rate. This is certainly true, and indeed, the question that the Reverend Bayes was primarily interested in was how to use evidence to update one's beliefs about such probabilities. This is a rich and important topic, but it is not the one we are examining here. Instead we are considering the simpler problem of using a set of known probabilistic quantities to infer another probabilistic quantity, the probability that the hypothesis is true in a particular instance, given some evidence.

In addition to knowledge of the prior probability of the hypotheses, *p*(*h*_1_) and *p*(*h*_2_), we also must know the probability of observing the evidence when each hypothesis it true. In our example, we need to know the probability of long hair when the person is a woman (for our example, *p*(*L*|*W*) or more generally *p*(*e*|*h*_1_)), and also the probability of long hair when the person is a man (*p*(*L*|*M*) or more generally, *p*(*e*|*h*_2_)). Here, too, there could be considerable uncertainty. However, as with the prior, we will treat these as quantities that are known, and proceed from there.

Using these quantities, we can plug them into Equation 1 to calculate *p*(*W*|*L*), the probability that the person your friend met is a woman given that the person had long hair. The expression below replaces the abstract variables *h*_1_ and *h*_2_ from Equation 1 with *W* and *M*, and replaces the abstract variable *e* with the *L* for long hair, to connect the various quantities in the expression to the relevant conceptual quantities in the example:

$$p(W|L)=\frac{p(W)p(L|W)}{p(W)p(L|W)+p(M)p(L|M)}$$

Let's plug in some actual numbers. If the overall probability of your friend meeting a woman, *p*(*W*), is 0.5; the probability of a woman having long hair *p*(*L*|*W*) is 0.8; and the probability of a man having long hair, *p*(*L*|*M*), is 0.3, then (relying on *p*(*M*) = 1 − *p*(*W*) = 0.5), we obtain:

$$p(W|L)=\frac{0.5\;*\;0.8}{0.5\;*\;0.8\;+\;0.5\;*\;0.3}=\frac{0.8}{0.8\;+\;0.3}=\frac{0.8}{1.1}=0.727$$

As an exercise, the reader can explore what happens to the result when one of the relevant quantities changes. What if *p*(*L*|*M*) goes down to 0.01? In a world where few men have long hair we get a much stronger conclusion. On the other hand, what if *p*(*L*|*M*) = 0.8? You should see that in this case we learn nothing about the person's gender from knowing the person has long hair. Now, what about the prior or base rate, *P*(*W*)? We have assumed that a person your friend might meet is equally likely to be a woman or a man, but what if instead *p*(*W*) is only 0.1—this might happen, for example, if the people your friend meets are all computer science majors. Using our initial values for the likelihoods *p*(*L*|*W*) = 0.8 and *p*(*L*|*M*) = 0.3, you should find that the posterior probability that the person is a woman is less than 0.3. If you neglected the base rate, you might overestimate this probability.

As a second exercise, the reader should be able to calculate *p*(*W*|*S*), the probability that a person your friend met is a woman given that the person had *short* hair, given specific values for *p*(*L*|*W*), *p*(*L*|*M*) and *p*(*W*). Use 0.8, 0.3, and 0.5 for these quantities. What gender should we guess to maximize the probability of being correct if we were told that a person your friend met had short hair? Assume for this example that each person either has short hair or long hair—that is, that short and long are mutually exclusive and exhaustive alternatives. As before, also assume that male and female are mutually exclusive and exhaustive alternatives.

Bayes' formula can easily be applied to cases in which the two hypotheses under consideration are the hypothesis that some proposition is true and the hypothesis that the proposition is false. For example, we might want to determine whether a person is French or not. In this case, our hypotheses could be ‘Person X is French’ and ‘Person X is not French,’ where no specific alternative hypothesis is specified. Here it is natural to use *h* for the positive case and $\bar{h}$ for the negative case, and to rewrite the formula as:

$$p(h|e)=\frac{p(h)p(e|h)}{p(h)p(e|h)+p(\bar{h})p(e|\bar{h})}$$

Given that *h* and $\bar{h}$ are assumed to be mutually exclusive and exhaustive, $p(\bar{h})=1-p(h)$, so we can also write our formula as:

(2) $$\begin{array}{l} p(h|e)=\frac{p(h)p(e|h)}{p(h)p(e|h)+(1-p(h))p(e|\bar{h})} \end{array}$$

It is also worth noting that the posterior probabilities sum to one: $p(h|e)+p(\bar{h}|e)=1$, so $p(\bar{h}|e)=1-p(h|e)$. Thus, the evidence simultaneously informs us about the posterior probability that *h* is true, and that *h* is false.

***Remark*:** Clearly, Bayes' formula only gives valid results if the quantities that go into the calculation are accurate. It would likely be wrong to assume that human perception always relies on the correct values of these quantities. One could propose that human perceivers rely on estimates of such quantities, and that these may differ from their actual values. A further point is that an experimenter might generate inputs according to a protocol that is not fully consistent with the knowledge perceivers rely on to make perceptual inferences. In that case, if the estimates perceivers rely on are not altered to match the protocol used in the experiment, the inferences could be invalid, and therefore not optimal under the conditions of the experiment. For example, a perceiver in a word identification experiment might rely on estimates of each word's probability of occurrence based on its frequency of occurrence in past experience. However, an experimenter might choose words from a word list without regard to their frequency. Under these conditions, use of a word's frequency to represent its probability of occurrence would be invalid. Many perceptual “biases” or “illusions” can be explained as resulting from the use of estimates of probabilistic quantities that may be valid (or approximately valid) in the real world, but are not valid within the context of the experiment. If such knowledge were wired into the connections among neurons in a perceiver's perceptual system, as it is assumed to be in the IA model, it might not be easily discarded and replaced with other values.

### Decision policies

So far, we have shown how to calculate a posterior probability, but we have not discussed what one might actually do with it. In many situations, we may simply want to take note of the posterior probability—in the case of our first example above, we might not wish to reach a definite conclusion, since the evidence is far from conclusive. However, often a choice between the alternatives is required. There are two possibilities that are often considered: one policy tries to pick the best response, that is, the one that maximizes the probability of being correct, while the other generates responses probabilistically, according to the posterior probability.

The first policy is called *maximizing*. This policy amounts to choosing the alternative with the largest posterior probability. Formally, we could write:

$$Choice=\text{argmax}(p(h_{1}|e),\;p(h_{2}|e))$$

where the **argmax** function returns the index of the hypothesis with the largest posterior probability. In our example, with the priors *p*(*W*) = 0.5, *p*(*L*|*W*) = 0.8 and *p*(*L*|*M*) = 0.3, we calculated that *p*(*W*|*L*) = 0.727 and it follows that *p*(*M*|*L*) = 0.273. Following this policy, then, we would conclude that the person is a woman given that the person has long hair.

The second policy is called *probability matching* or just *matching*. Under this policy, decision makers' choices would vary from trial to trial with the same evidence, but would occur with a probability that matches the posterior probability. Formally, we would write this as:

$$p(Choice=i)=p(h_{i}|e)$$

One of these two policies is better than the other, in the sense that one maximizes the probability of choosing the correct answer. If you would win a dollar for guessing right and loose a dollar for guessing wrong, which of these policies should you chose? Surprisingly, in many cases, the behavior of humans and other animals appears closer to matching rather than maximizing, but there are situations in which people clearly do maximize (Green et al., 2010). There are worse policies than matching. One such policy sometimes used in explicit outcome guessing tasks by children around age five is to alternate choices from one trial to the next, regardless of the probability of each of the two outcomes, and even when the trial sequence is completely random (Derks and Paclisanu, 1967).

### BAYES' theorem: bayes' formula follows from the definition of conditional probability

So far, we have used Bayes' formula without considering why it is true. Here, we will show that the validity of the formula follows from the definition of conditional probability. We have already used the concept of conditional probability. Here we will review its definition and then use it to derive Bayes' formula.

The conditional probability of some event *a* given some other event *b*, written *p*(*a*|*b*), is defined as the ratio of the probability of both *a* and *b*, *p*(*a*&*b*) to the probability of *b*, *p*(*b*):

$$p(a|b)=\frac{p(a\text{\&}b)}{p(b)}$$

The definition can be read as defining conditional probability *p*(*a*|*b*) as the proportion of the times when *b* occurs that *a* also occurs. Let's relate this to our case, letting *e* correspond to *a* and *h* correspond to *b*:

(3) $$p(e|h)=\frac{p(e\text{\&}h)}{p(h)}$$

In our case, if 50% of the people your friend might meet are women, and 40% of the people your friend might meet are women with long hair, then the probability of long hair given that the person is a woman—or equivalently, the proportion of women who have long hair—would be 0.4/0.5 = 0.8, the value we already used in our example.

Now we can also use the definition of conditional probability to express *p*(*h*|*e*), letting *e* correspond to *b* and *h* correspond to *a*:

(4) $$p(h|e)=\frac{p(e\text{\&}h)}{p(e)}$$

Bayes' formula can now be derived from the fact that *p*(*e*&*h*) occurs in the definition of both *p*(*e*|*h*) and *p*(*h*|*e*). To derive it, we multiply both sides of Equation 3 by *p*(*h*) to obtain:

p(e&h)=p(h)p(e|h)

For our example, this corresponds to the fact that the proportion of people who have long hair and are women is equal to the proportion of all people who are women, times the proportion of women who have long hair.

We can now replace *p*(*e*&*h*) in Equation 4 with *p*(*h*)*p*(*e*|*h*) to obtain:

$$p(h|e)=\frac{p(h)p(e|h)}{p(e)}$$

This can be stated: the probability of some hypothesis *h* being true given some evidence *e* is equal to the prior probability of the hypothesis, *p*(*h*), times the probability of the evidence, given the hypothesis, divided by the overall probability of the evidence *p*(*e*).

It remains only to note that the denominator, the probability of the evidence *p*(*e*), is equal to the probability of the evidence occurring when the hypothesis is true plus the probability of the evidence occurring when the hypothesis is false, $p(e\text{\&}h)+p(e\text{\&}\bar{h})$. That is, the total probability of situations in which *e* is true is the sum of the probabilities of two situations, one in which *e* is true and the hypothesis *h* is also true, and another in which *e* is true and the hypothesis is false. This exhausts the cases in which *e* is present, given that *h* must either be true or not. Using the fact that *p*(*a*&*b*) = *p*(*b*)*p*(*a*|*b*) twice more, applying it to both *p*(*e*&*h*) and to p(e&h¯), we finally obtain:

$$p(h|e)=\frac{p(h)p(e|h)}{p(h)p(e|h)+p(\bar{h})p(e|\bar{h})}$$

and from $p(\bar{h})=1-p(h)$, we can then obtain Equation 2. Of course the same all works out for cases in which we have two mutually exclusive and exhaustive hypotheses called *h*_1_ and *h*_2_ as in the version shown in Equation 1, as well.

**Figure 1:**
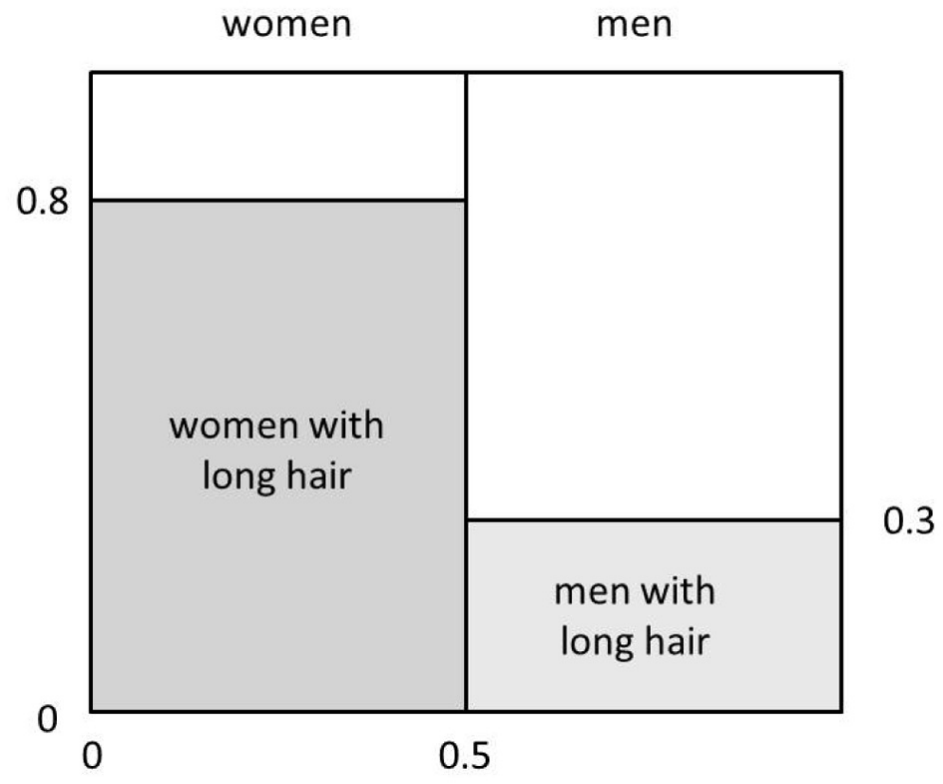
**Graphical depiction of posterior probability based on relative area**.

Figure 1 gives a graphical representation of the posterior probability of a hypothesis constructed by partitioning a square with sides of length 1. We use the horizontal dimension to partition the square into two parts by drawing a vertical line at *x* = *p*(*W*), so that the area to the left of the line corresponds to the overall probability that a person your friend might meet would be a woman and the remaining area corresponds to the probability that the person your friend might meet would be a man. Restating, the areas of these two parts correspond to *p*(*W*) and *p*(*M*), respectively. Then, we partition the region corresponding to women into two parts along the vertical axis at the point *y* = *p*(*L*|*W*). This divides the total probability that the person is a woman into two parts, one corresponding to the probability that the person is a woman and has long hair, and one corresponding to the probability that the person is a woman and does not have long hair. Likewise, we partition the region corresponding to men into two parts along the vertical axis at the point *y* = *p*(*L*|*M*). This gives us two more rectangles, one whose area corresponds to the probability that the person is a man and has long hair, and the other corresponding to the probability that the person is a man and does not have long hair. The area of each resulting rectangle is a joint probability as well as the product of a prior and a conditional probability. The posterior probability *p*(*W*|*L*) is the ratio of the area of the rectangle corresponding to women with long hair to the area corresponding to all persons with long hair, which in turn corresponds to the sum of the areas of the two shaded rectangles.

To fix your understanding of these ideas, you could draw an approximate version of this figure for the case in which (i) the overall probability that a person your friend might meet is a woman is 0.25; (ii) the probability of a woman having long hair is 0.75; and (iii) and the probability of a man having long hair is 0.25. Inspecting the relevant subrectangles within the unit rectangle, you should be able to estimate the probability that the person your friend meets is a woman, given that the person has long hair. You would do this by noting the area corresponding to the probability of being a woman and having long hair, and comparing that to the area corresponding to the probability of being a man and having long hair. Given that these areas are about equal, what is the probability that a person with long hair is a woman in this case?

### Multiple alternative hypotheses

**Figure 2:**
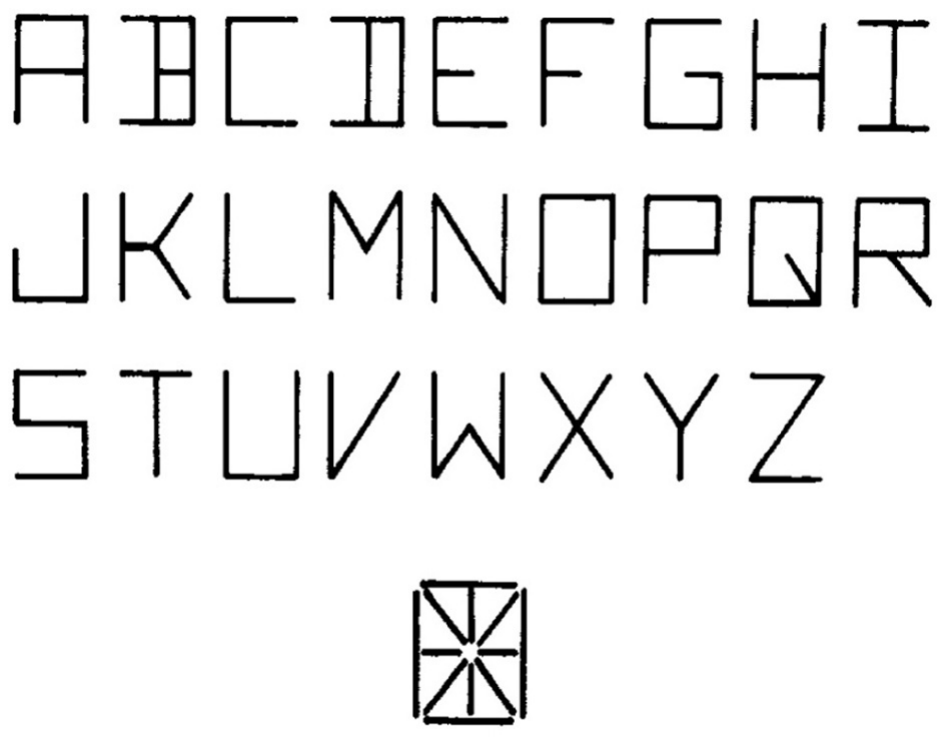
**The line segments used in the Rumelhart and Siple font and the letters composed from these segments.** From Rumelhart and Siple (1974). Reprinted with permission.

We have thus far considered cases in which there are only two possible hypotheses, for example, either the person my friend met was a woman or the person was a man. Now let us suppose we have many alternative hypotheses {*h*_*i*_}, and we are trying to determine the posterior probability of each given some evidence *e*. One example arises if we are trying to determine the identity of a letter given one of its features. For example, in the font used by Rumelhart and Siple (1974), and in the MIA model, one of the features (which we will call *F*_*ht*_) is a horizontal line segment at the top of a letter-feature block (See Figure 2). Some letters have this feature, and others do not. For example, the letter *T* has it and the letter *U* does not. Treating these statements as absolutes, we could state *p*(*F*_*ht*_|*T*) = 1 and *p*(*F*_*ht*_|*U*) = 0. However, let us allow for the possibility of error, so that with a small probability, say 0.05, feature values will be registered incorrectly. Then *p*(*F*_*ht*_|*T*) = 0.95 and *p*(*F*_*ht*_|*U*) = 0.05. Now, suppose we want to calculate *p*(*T*|*F*_*ht*_). For each letter, *l*_*i*_ we would need to know *p*(*F*_*ht*_|*l*_*i*_) and we would also need to know the prior probability of occurrence of each letter as well. Given this information, the overall formula for the posterior probability now becomes:

$$p(T|F_{ht})=\frac{p(T)p(F_{ht}|T)}{\sum_{i^{\prime }}p(l_{i^{\prime }})p(F_{ht}|l_{i^{\prime }})}$$

Note that the summation^2^ in the denominator runs over all possible letters, including *T*. In general, the probability that a particular hypothesis *h*_*i*_ is correct given a specific element of evidence *e* can be written:

$$p(h_{i}|e)=\frac{p(h_{i})p(e|h_{i})}{\sum_{i^{\prime }}p(h_{i^{\prime }})p(e|h_{i^{\prime }})}$$

The indexing scheme is potentially confusing: Here and elsewhere, we use a bare single letter such as *i* to index a specific item or hypothesis of interest and a primed version of the same letter such as *i*′ to index all of the items or hypotheses, including *i*.

It is useful at this point to introduce the notion of a *multinomial random variable*, defined as a random variable that can take any one of *n* discrete values, such as letter identities. This generalizes the notion of a binary random variable, which is one that can take either of two possible values (such as *true* or *false* or *man* or *woman*). We can think of the identity of a given letter, for example, as a multinomial random variable having one of 26 possible values. The name of the multinomial interactive activation model is based on the idea that (in letter perception) the task of the perceiver is to infer the correct values of several such multinomial variables—one for the identity of each of the four letters in a letter string, and one for the identity of the visually presented word—from visual input. For now, we are working with the simpler case of attempting to set the value of a single multinomial variable corresponding to a single letter identity.

The prior associated with a multinomial random variable is the vector of prior probablities *p*(*h*_*i*_). Under the assumption that the hypotheses are mutually exclusive and exhaustive, the sum of the *p*(*h*_*i*_) should be equal to 1. In the specific case of possible letter identities, given that there are 26 letters, there are only 25 independent letter probabilities, since the probability of the last one must be equal to 1 minus the sum of the probabilities of all of the others. In general, if there are *N* mutually exclusive possibilities, there are only *N* − 1 degrees of freedom in the values of their prior probabilities^3^.

Even when there are multiple possibilities, we note that if only one of these hypotheses is of interest—when, say, we are interested in knowing whether a given letter is a *T* or not—all of the other possibilities can be lumped together and we have:

$$p(T|F_{ht})=\frac{p(F_{ht}|T)p(T)}{p(F_{ht}|T)p(T)+\sum_{i^{\prime }\;\neq \;T}p(F_{ht}|l_{i^{\prime }})p(l_{i^{\prime }})}$$

where the summation in the denominator runs over all possible letters other than T of terms corresponding to the product of the prior and the likelihood. This is a generalization of Equation 2, previously given, with ∑_*i*′ ≠ *T*_*p*(*F*_*ht*_|*l*_*i*′_)*p*(*l*_*i*′_) playing the role of $p(F_{ht}|\bar{T})p(\bar{T})$^4^.

It is also worth noting that in some situations, we may want to include the possibility that the observed input arose from some unknown cause, outside of a specifically enumerated set. For example, some feature arrays that appear in a letter perception experiment might have been generated from something other than one of the known letters. We can include this possibility as an additional hypothesis, if we also provide the probability that the feature value arises from this other cause. In this case the sum of the probabilities of the enumerated causes is less than one, with the other causes consuming the remainder of the total probability. Then we can write Bayes Formula as:

$$p(h_{i}|e)=\frac{p(h_{i})p(e|h_{i})}{\sum_{i^{\prime }}p(h_{i^{\prime }})p(e|h_{i^{\prime }})+p(o)p(e|o)}$$

where *p*(*o*) is the prior probability for all other causes and *p*(*e*|*o*) is the probability of the evidence arising from any of these other causes. In psychological models, e.g., the Logogen model of word recognition (Morton, 1969), or the generalized context model of categorization (Nosofsky, 1984), the elements of the expression *p*(*o*)*p*(*e*|*o*) are not separately estimated, and are lumped together in a constant.

### Multiple elements of evidence and conditional independence

In general, when we are attempting to recognize letters or other things, there may be more than one element of evidence (e.g., more than one feature) at a time. How can we deal with such situations? A first step is to generalize Bayes' formula by using a likelihood term that encompasses all of the evidence. For example, we might have evidence that there is a horizontal feature across the top of a feature array and a vertical segment down the middle. We could then make use of expressions such as *p*(*F*_*ht*_&*F*_*vm*_|*T*) to represent the probability of observing both of these features, given that the letter in question is *T*.

A problem that arises here is that the number of possible combinations of elements of evidence can grow large very quickly, and it becomes intractable to assume that a perceiver knows and represents all of these probabilities. Luckily, there is a condition under which the computation of the values of such expressions becomes very simple. This condition is known as *conditional independence*, which can be defined for two or more events with respect to some other, conditioning event. For two events, conditional independence is defined as follows:

***Definition of Conditional Independence***. Elements of evidence *e*_1_ and *e*_2_ are conditionally independent given condition *c* if the probability of both pieces of evidence given *c*, *p*(*e*_1_&*e*_2_|*c*), is equal to the product of the separate conditional probabilities *p*(*e*_1_|*c*) and *p*(*e*_2_|*c*) for each element of the evidence separately.

We can generalize this to an ensemble of any number of elements of evidence *e*_*i*_ and express the relationship succinctly: Conditional independence of an ensemble of *n* elements of evidence *e*_*i*_ given some condition *c* holds when:

$$p(e_{1}\text{\&}e_{2}\text{\&}\ldots \;\text{\&}e_{n}|c)=\prod_{j}p(e_{j}|c).$$

Considering our example, we can consider the presence of a horizontal across the top, *F*_*ht*_, and the presence of a vertical down the middle, *F*_*vm*_. These would be conditionally independent given that the underlying letter was in fact intended to be a *T* if it were true of the world that error entered into the registration of each of these two features of the letter T independently.

We can now write a version of our formula for inferring posterior probabilities under the assumption that conditional independence holds for all elements of evidence *e*_*j*_ conditioned on all of the hypotheses *h*_*i*_:

$$p(h_{i}|e_{1}\text{\&}e_{2}\text{\&}\ldots \;\text{\&}e_{n})=\frac{p(h_{i})\prod_{j}p(e_{j}|h_{i})}{\sum_{i^{\prime }}p(h_{i^{\prime }})\prod_{j}p(e_{j}|h_{i^{\prime }})}$$

We are still relying on many probabilistic quantities, but not as many as we would have to rely on if we separately represented the probability of each feature combination conditional on each hypothesis.

***Remark*:** Clearly, the assumption of conditional independence is unlikely to be exactly correct. However, it is hard to imagine proceeding without it. One way of alleviating the concern that relying on this assumption will lead us astray is to note that in cases where the occurrence of elements of evidence is highly correlated (even after conditioning on hypotheses), we might treat these elements as a single element, instead of as separate elements. Maybe that is what features are: clusters of elements that have a strong tendency to co-occur with each other. Another response to this situation would be to note that any explicit probability model involving sets of explicit hypotheses and elements of evidence is unlikely to be exactly correct for naturalistic stimuli. Words spelled using letters and their features as in the Rumelhart font are not really natural stimuli, since these items actually do consist of discrete units (letters) and these in turn consist of independent sub-units (letter features). This allows for the possibility of validly characterizing displays of such features in terms of a process in which conditional independence of features holds exactly. A learned, implicit probability model of the kind embodied in a Deep Belief Network (Hinton and Salakhutdinov, 2006) is likely to be a better model for naturalistic stimuli.

#### A generative model of feature arrays

Consider the following description of how displays of letter features registered by a perceiver might be generated. An experimenter selects a letter to display from the alphabet with probability *p*(*l*_*i*_), which for now we will take to be simply 1/26 for each letter, and then generates a feature array as follows. Each letter has a set of correct feature values. For example, for *T*, the feature *F*_*ht*_ is present, the feature *F*_*vm*_ is present, and the feature *F*_hb_, a horizontal line across the bottom, is absent (for simplicity, we will just consider these three features for now). However, when the actual feature array is generated, there is some small probability that each feature will not be generated correctly. The correctness of each feature is separately determined by an independent random process, e.g., by rolling a 20-sided die with a spot on just one side. If the spot comes up, the incorrect value of the feature is displayed. If it does not, the feature is generated correctly. The die is rolled once for each feature, and we are expressly assuming that the outcome of each roll is independent of the outcomes of all other rolls.

The above is a simple example of a generative model. If features were generated according to this process, then the probabilities of features are conditionally independent, given the letter identities. Note that if the generative process usually works perfectly and correctly generates all the correct features, but occasionally hiccups and gets all the features wrong at the same time, the elements of the evidence would not be conditionally independent. Note also that conditional independence can hold if the probability of feature perturbation is different for different features; this is likely if we think of the perturbation as occurring within the visual system, so that some features are more likely to be mis-registered than others, due to differences in their size, retinal position, or other factors.

Now, the true process generating feature arrays may not be exactly as described, just as the prior and likelihood values used may not be exactly accurate. However, a generative model in which feature values are perturbed independently can be treated as an assumption about the actual generative process, or alternatively it can be treated as an assumption about the model of the generative process that is utilized by the perceiver in a letter perception experiment. Such a model could be false, or only approximately true, and still be used by a perceiver. A further possibility is that the true model used by the perceiver is more complex, but that the assumption that the perceiver uses such a model provides a good approximation to the true model being used by the perceiver.

### The support for an hypothesis and the luce choice rule

It will be helpful in our later development to write an expression we will call the Support (*S*_*i*_) for a given alternative hypothesis *h*_*i*_, given a set of elements of evidence {*e*} = {*e*_1_, *e*_2_, …} as follows:

$$S_{i}=p(h_{i})\underset{j}{\prod }p(e_{j}|h_{i})$$

For our example, the *h*_*i*_ correspond to the different possible letter hypotheses and the *e*_*j*_ correspond to the elements of the evidence. We will describe this overall support as consisting of the product of two terms, the prior *p*(*h*_*i*_) and the likelihood *p*(*e*|*h*_*i*_), which under the generative model described above is equal to the product of terms that might be called the element-wise likelihoods of each element of the evidence.

With this expression for the support of hypothesis *i* in the presence of evidence {*e*}, we can write Bayes' formula as:

(5) $$p(h_{i}|\{e\})=\frac{S_{i}}{\sum_{i^{\prime }}S_{i^{\prime }}}$$

As before, *i*′ is an index running over all of the alternative hypotheses, including hypothesis *i*. Readers familiar with the Luce (1959) choice rule will notice that this expression corresponds to Luce's rule, with the *S*_*i*_ corresponding to the response strengths associated with the different choice alternatives.

As an exercise, consider a letter microworld with just the three features we have considered so far and just the letters *T*, *U* and *I*. Assume that according to the generative model, each letter is equally likely *p*(*T*) = *p*(*U*) = *p*(*I*) = 1/3. Regarding the features, we follow a policy used in the original IA model and carried over in the multinomial IA model: we explicitly represent the absence of a feature as an element of evidence, just like the presence of a feature. Thus, there are six possible elements of evidence or feature values relevant to identifying letters: a feature can be present or absent, for each of the three possible features.

To proceed with our exercise, the probability of each possible feature value (present or absent) is given for each of the three possible feature dimensions of each letter in Table 1. Here *h* stands for a high probability (let's say 0.95) and *l* for a low probability (0.05). Features cannot be both present and absent, so *l* = 1 − *h*. Assuming actual features are generated in a conditionally independent manner, we can then ask, what is the probability that the underlying letter was a T given that the following evidence {*e*} is available: Horizontal at top *present*, Vertical at middle *absent*, Horizontal at bottom *absent*. Although these features do perfectly match the high-probability values for the letter *T*, the letter is more likely to be a *T* than a *U* or an *I*. See if you can verify this. Using the two equations above, along with Table 1 and the specific numerical values given in this paragraph, you should be able to obtain an explicit probability for *p*(*T*|{*e*}). You should also be able to express simply why T is more probable that U or I given the available evidence.

**Table 1:** **Probability that features take given values in the Letters T, U, and I**.

**Letter**	**Feature**					
	**Horiz. at Top**		**Vert. thru Middle**		**Horiz. at Bottom**	
	**Present**	**Absent**	**Present**	**Absent**	**Present**	**Absent**
T	h	l	h	l	l	h
U	l	h	l	h	h	l
I	h	l	h	l	h	l

*h in the table corresponds to a high probability, such as 0.95, and l corresponds to a low probability, such as 0.05*.

One additional issue may now be considered. We may ask, what happens if we are not told about one of the elements of the evidence? For example, we are told that the horizontal bar across the top is present and the vertical bar down the center is present but we simply are not told about the horizontal bar across the bottom (perhaps something is blocking our view of that feature in a perceptual display, for example). We would simply use those elements of evidence that we do have, and exclude the elements that are unspecified. Our existing expression already captures this policy implicitly, since when an element of evidence is missing it simply does not show up in the ensemble of elements *e*_*j*_. However, it will prove useful to capture this case by elaborating the expression for S above to include explicit information specifying whether particular items of evidence are or are not present. A nice way to do this is to have a binary vector indicating whether the element of evidence is present or not. We have six possible elements of evidence in our example, as enumerated above. If we are given Horizontal at Top *present*, Vertical thru Middle *absent*, this vector would become: *v* = 1 0 0 1 0 0. Then we would obtain the same results as before by writing *S*_*i*_ as follows:

(6) $$S_{i}=p(h_{i})\underset{j}{\prod }p{\left(e_{j}|h_{i}\right)}^{v_{j}}$$

Where ∏_*j*_ represents the product over all possible elements, and *v*_*j*_ is equal to 1 for elements of evidence that are present, or 0 otherwise. Note that elements that are absent have no effect since for any non-zero *x*, *x*^0^ = 1, and for all *p*, *p* · 1 = *p*.^5^ Note that the model we use here distinguishes between evidence of absence (“No horizontal bar is present at the bottom of the feature array”) and the absence of evidence (“We do not know whether or not a bar is present at the bottom of the feature array”). In many cases, it is useful to distinguish between these two situations.

***Remark: Using *p*(e*|*h)* to infer *p(h|e)*** It is worth noting that we use knowledge of the probability of evidence given a hypothesis to infer the probability of a hypothesis given evidence. At first, this may seem counter-intuitive. Why don't we just store the value of *p*(*h*|*e*), rather than always having to compute it? A similar counter-intuition arises in thinking about the “bottom-up” support for letter hypotheses by feature evidence. One might think that the effect of a feature's presence on the probability of a letter should depend on the probability of the letter given the feature, and not the other way around. The resolution of this counter-intuition depends on noticing that the posterior probabilities are not directly defined in the generative model, while the prior and the *p*(*e*|*h*) terms are. Indeed, the posterior probability that a hypothesis is true depends on the entire ensemble of quantities in the generative model and the particular ensemble of elements of evidence that may be present, while the *p*(*h*) and *p*(*e*|*h*) values can be stable and independent. To contemplate this in a specific context, let us return to the question of the probability that a person is a woman, given that she has long hair. This quantity depends on three other quantities: the overall probability that a person is a woman; the probability that a woman has long hair; and the probability that a man has long hair. Each of these quantities can be changed independently, without affecting the others, while the probability that a person with long hair is a woman depends on all three. In short, in many contexts at least, it makes sense that we use *p*(*h*) and *p*(*e*|*h*) to compute *p*(*h*|*e*).

### Summary: generalized version of bayes formula

To summarize the above development, the generalized version of Bayes formula for the posterior probability of hypothesis *h*_*i*_, for *i* = 1, …, *n* mutually exclusive hypotheses and *j* = 1, …, *m* possible conditionally independent elements of evidence is:

$$\begin{array}{l} p(h_{i}|e)=\frac{S_{i}}{\sum_{i^{\prime }}S_{i^{\prime }}}, \end{array}$$

where *S*_*i*_ stands for the support for hypothesis *h*_*i*_, defined as:

$$\begin{array}{l} S_{i}=p(h_{i})\underset{j}{\prod }p{\left(e_{j}|h_{i}\right)}^{v_{j}} \end{array}$$

## Calculating posterior probabilities with connectionist units using the softmax and logistic functions

**Figure 3:**
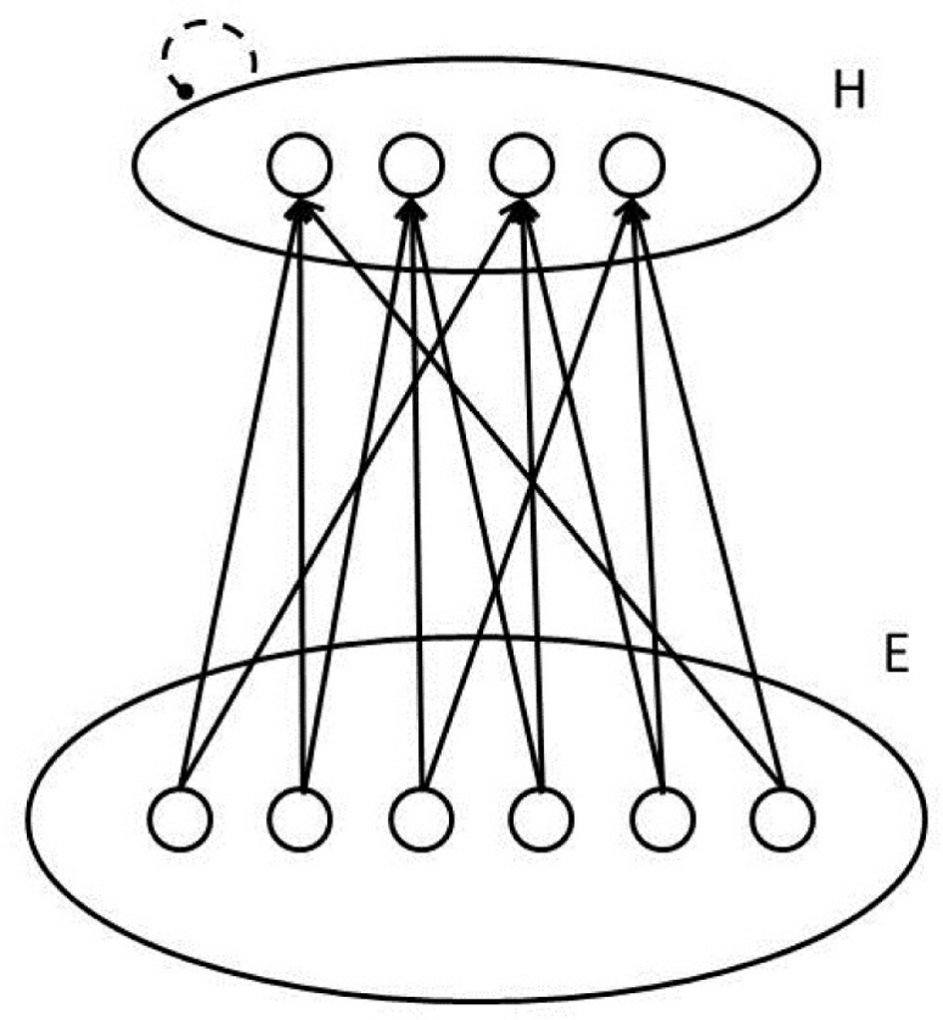
**Sketch of a pool of units that can calculate posterior probabilities of patterns represented on its inputs using the softmax function.** Dashed line signifies lateral inhibition to normalize the activations of units in the pool.

We now develop the idea that the posterior probability calculation just presented can be computed by a group of connectionist processing units, using a function called the **softmax** function. The neural network is illustrated in Figure 3. In this network, each unit corresponds to an hypothesis *h*_*i*_, and has a bias term *b*_*i*_, as well as incoming connections from units outside the ensemble. Each of these outside units indexed by *j* stands for a possible element of evidence. When the element of evidence is present, the unit will have an activation value *a*_*j*_ equal to 1; when it is absent, its activation will be 0. Each connection to a hypothesis unit from an evidence unit will have a strength or weight represented by the variable *w*_*ij*_.

Concretely, pursuing our example, the units in the pool could correspond to possible letters, each unit's bias term could reflect a perceiver's bias to think the input contains the given letter, and the connection weights could reflect the perceiver's tendency to think the hypothesis is more (or less) likely, when the corresponding element of evidence is present. The pool described corresponds to one of the pools of letter level units in the MIA model, although we are considering just one such pool in isolation for now, without additional input from the word level.

In our network, as in most neural networks, each unit computes a summed or net input that reflects both its bias and the weighted sum of activations of other units:

$$net_{i}=b_{i}+\sum_{j}w_{ij}a_{j}$$

We will now see that if we set the weights and biases to appropriate values, then apply the **softmax** function defined below, the output of the function, represented here as ρ_*i*_, will be equal to the posterior probability of the letter the unit stands for, as expressed by the generalized Bayes formula.

The **softmax** function is:

$$\rho_{i}=\frac{e^{net_{i}}}{\sum_{i}^{\prime }e^{net_{i^{\prime }}}}$$

The reader should already be able to see that the softmax has some relationship to the generalized Bayes formula. Indeed, as we shall discuss, the expressions *e*^*net*_*i*_^ and *e*^*net*_*i*′_^ correspond to the expressions for *S*_*i*_ and *S*_*i*′_ in that equation.

The essential idea is that the bias term and the weights will be chosen to correspond to the logarithms of the quantities that are multiplied together to determine the *S*_*i*_ terms. Using the logs of these quantities, we add rather than multiply to combine the influences of the prior and the evidence. The resulting net input term corresponds to the log of the *S*_*i*_ terms defined above. We then reverse the logarithmic transformation at the end of the calculation, using the exponential function.

**Figure 4:**
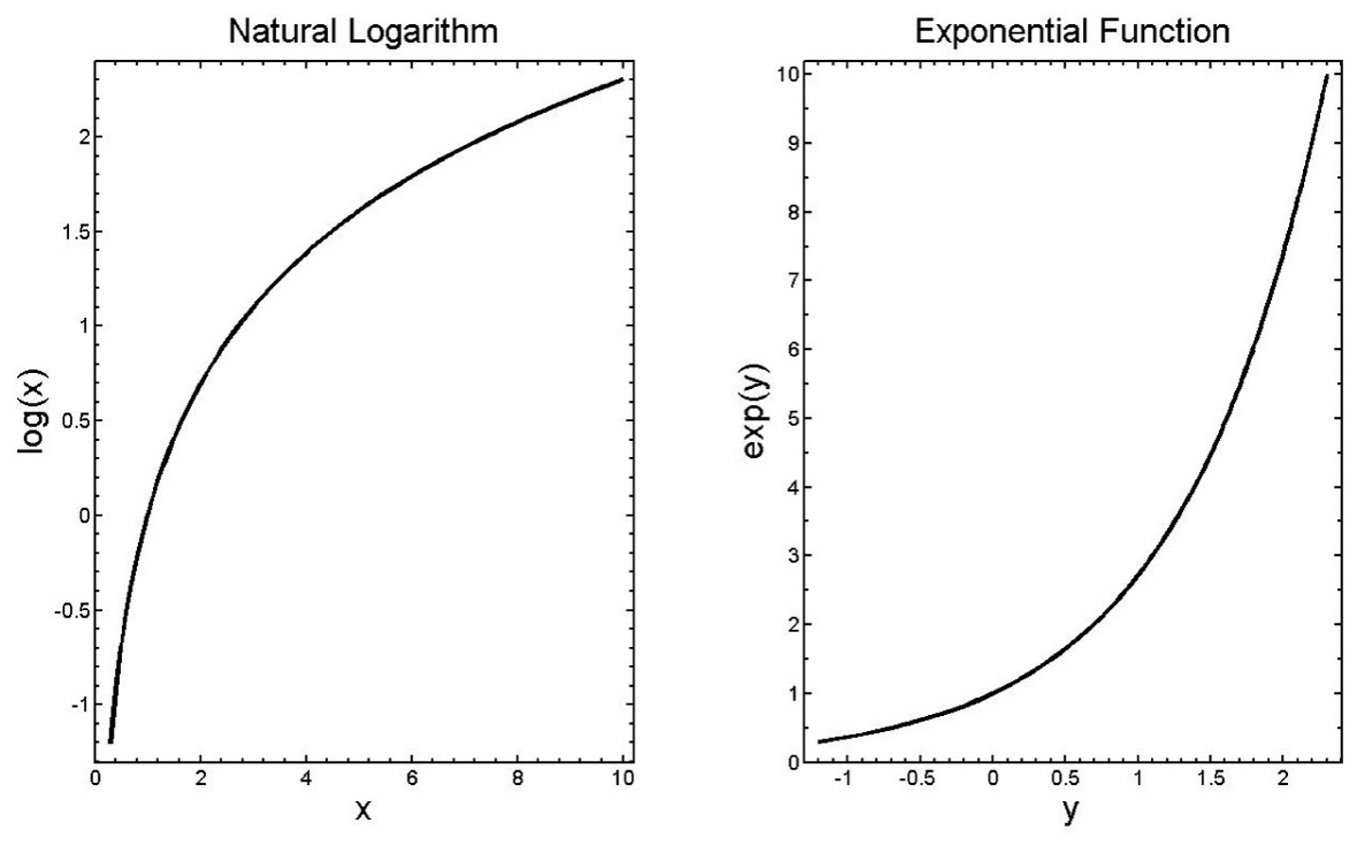
**The log and exponential functions**.

The analysis relies on several facts about the log and exponential functions that we now review. First, the function *y* = log(*x*) is defined as the function that produces, when applied to its argument *x*, a number *y* such that *e*^*y*^ = *x*. Note that log is used here to correspond to the natural logarithm, sometimes written log_*e*_ or ln. The exponential function of *y*, *e*^*y*^ corresponds to the number *e* taken to the power *y*, and is sometimes written exp(*y*). Given these definitions, it follows that log(*e*^*y*^) = *y* and *e*^log(*x*)^ = *x*. The graphs of the log and exp functions are shown in Figure 4.

The second important fact is that the log of the product of any number of quantities is the sum of the logs of the quantities:

log(a · b · c · …)=log(a)+log(b)+log(c)+…

Similarly, the log of the ratio of two quantities is equal to the difference between the logs of the quantities:

log(a/b)=log(a)−log(b)

Finally, the log of a quantity to a power is that power times the log of the quantity:

$$\log(a^{b})=b\log(a)$$

There are also useful related facts about exponentials, namely *e*^(*a* + *b* + *c* + …)^ = *e*^*a*^ · *e*^*b*^ · *e*^*c*^ · …; $e^{\left(a-b\right)}=\frac{e^{a}}{e^{b}}$; and *e*^(*a* · *b*)^ = (*e*^*a*^)^*b*^^6^.

With this information in hand, we consider the expression we previously presented for *S*_*i*_, the support for hypothesis *i*:

$$S_{i}=p(h_{i})\prod_{j}p{\left(e_{j}|h_{i}\right)}^{v_{j}}$$

Taking logs, we see that:

$$\log(S_{i})=\log(p(h_{i}))+\sum_{j}v_{j}\log(p(e_{j}|h_{i}))$$

It should now be apparent that the net input as described above would correspond to the log of the support for the hypothesis represented by the unit if: (a) the value of the bias term were set to correspond to the log of the prior probability of the hypothesis; (b) each incoming weight were set to correspond to the log of the probability of the corresponding element of the evidence given the hypothesis; and (c) the activation of the external unit sending activation through the weight were to be equal to 1 when the evidence is present, and 0 otherwise. Stated succinctly in terms of defined quantities:

$$net_{i}=\log(S_{i})\;if\;b_{i}=\log(p(h_{i})),\;w_{ij}=\log(p(e_{j}|h_{i})),\text{ and }a_{j}=v_{j}.$$

Now it should be clear that applying the softmax function:

$$\rho_{i}=\frac{e^{net_{i}}}{\sum_{i^{\prime }}e^{net_{i^{\prime }}}}$$

should set the value of the variable ρ_*i*_ to be equal to the posterior probability of hypothesis *i* given the set of elements of the evidence *e*_*j*_ as long as *net*_*i*_ corresponds to log(*S*_*i*_) for all *i*, since *e*^log(*x*)^ = *x*, as noted above. Substituting log(*S*_*i*_) and log(*S*_*i*′_) into the softmax function where we find *net*_*i*_ and *net*_*i*′_ we will clearly obtain our generalized Bayes formula.

Thus, the neural network in Figure 3, employing the softmax function, calculates posterior probabilities by relying on a non-linear but monotonic function (the exponential function) of the sum of a set of terms, one for the prior probability of the hypothesis and one for each of the elements of the evidence.

***Why sums rather than products?*** One might be inclined to ask at this point, why should neural network modelers even bother computing net inputs as additive quantities? Why not compute the posterior probabilities more directly, without ever taking logs? The answer may in part be historical: the original model neuron introduced by McCulloch and Pitts (1943) summed weighted inputs, and if they exceeded a threshold the neuron's output was set to 1; otherwise the output was 0. This was intended to mimic both real neurons (which fire action potentials if their state of depolarization reaches a critical level) and logic gates (devices then send out a 1 or a 0 based on some logical function of their inputs). The logistic function discussed below, a close relative of the softmax function, was adopted for use in neural network models because it produced a graded rather than a discrete response, and could be differentiated. Only later did the connection to probability become apparent [first reflected, to my knowledge, in Hinton and Sejnowski (1983)]. But what about the brain itself? It is common to treat synaptic currents as being summed to determine the neuron's potential, which in turn determines its firing rate according to a non-linear function. It is possible that addition may be more robust and easier to implement in neurons than multiplication, especially when small probabilities are involved, since noise affecting such quantities can drastically distort the results of multiplying products, and in any case the computations are just as valid when conducted using addition of logarithms rather than multiplication, as long as we have a non-linear activation function like softmax to convert the influences back. Some further relevant observations are provided below.

### Maximizing and matching using the neural network

We can imagine a number of policies we might employ in using the ρ_*i*_ values as a basis for overt responding. One policy would be to choose the alternative with the largest value of ρ_*i*_; this corresponds to maximizing. Matching would occur if we were to choose alternatives with probability equal to the value of ρ_*i*_. A gradient of possibilities between these extremes can be obtained by introducing a parameter usually called temperature, following the analogy to statistical physics introduced into neural networks research by Hinton and Sejnowski (1983). This usage corresponds to the analogy from physics, in which the temperature determines the degree of randomness in the behavior of elements of the system. In this version of the formula, our expression now becomes:

$$\rho_{i}(T)=\frac{e^{net_{i}/T}}{\sum_{i^{\prime }}e^{net_{i^{\prime }}/T}}$$

Our previous case corresponds to the situation in which *T* = 1. We can now imagine a policy in which we choose each alternative with probability ρ_*i*_(*T*), for different values of the *T* parameter. As *T* becomes small, the largest net input term strongly dominates, and in the limit as *T* → 0 our policy converges on maximizing, since ρ_*i*_(*T*) will approach 1 for the unit with the largest net input and will approach 0 for all other units. As *T* becomes large, the ρ_*i*_(*T*) will all approach 1/*N* where *N* is the number of alternatives, corresponding to random guessing.

*Example*. The softmax function can be used to model response choice probabilities in many situations, under a matching assumption, where the ρ_*i*_ correspond to choice probabilities. One case where the model provided an excellent fit arose in an experiment by Salzman and Newsome (1994). Here a monkey received a visual motion stimulus, corresponding to evidence favoring a particular alternative direction out of eight alternative motion directions. On some trials, the monkey also received direct electrical stimulation of neurons representing motion in a particular direction (treated in the model as another source of conditionally independent evidence). The monkey's choice behavior when both sources of evidence were presented together corresponded well to the predictions of the model. The experimenters estimated quantities corresponding to the bias terms and weights used in the softmax formulation. Although they did not mention Bayesian ideas, these terms could be treated as corresponding to logarithms of the corresponding Bayesian quantities.

***Lateral inhibition and effects of noise in the net input***. The denominator of the softmax function can be seen as expressing a particular form of lateral inhibition, in that strong support for one alternative will reduce the value of ρ_*i*_ for another. Some readers may notice that the inhibitory influence a unit exerts on others depends on its net input term (specifically, *e*^*net*_*i*/*T*_^), whereas it is natural to think of the ρ_*i*_ as corresponding to the activations of the units for different alternatives. In most neural network models, units are usually thought to transmit their activation value, not their net input, both to exert excitatory and inhibitory influences. Do units use one variable for mutual inhibition and another to influence outside units? It is certainly a possibility. A computation of this kind could certainly be carried out, say, if the units in our networks corresponded to columns of neurons, in which some engaged in lateral inhibitory interactions while others sent excitatory signals to neurons in other pools. Also, it may be worth noticing that in practice, an iterative computational procedure in which the net input terms build up gradually and the denominator relies on the ρ_*i*_ terms instead of the *e*^*net*_*i*_^ terms should converge to the same result, as in the REMERGE model of memory trace activation (Kumaran and McClelland, 2012).

It is also possible to view the softmax function as describing the outcome of a simple winner-take-all process. Suppose we simply allow each unit to compute its net input, subject to noise, and adopt the policy of choosing as our response the unit with the largest net input. If the noise is very small, and the weights and biases correspond to the probabilistic quantities above, then by choosing the unit with the largest net input we will always be maximizing the posterior probability. On the other hand if the noise is sufficiently large, the net input will be effectively swamped by the noise, and choosing the unit with the largest net input will correspond to random responding. With an intermediate amount of noise, the process just described approximates choosing alternatives with probability ρ_*i*_(*T*) as calculated by the softmax function, for some value of the parameter *T* that depends on the amount of noise. In fact, if the noise affecting each unit is identically distributed according to a distribution called the extreme value distribution, then the choice probabilities will match those described by the softmax function exactly (Train, 1993). For those not familiar with the extreme value distribution, it is somewhat different from the Gaussian distribution, in that it is slightly skewed, but the shape is not drastically different from Gaussian, and simulations using Gaussian noise yield similar results to those expected using the extreme value distribution. The best characterization of noise in real neural populations is a matter of ongoing investigation, and it may not exactly match either the Gaussian or the extreme value distribution. In the absence of complete consensus, it seems reasonable to treat the noise in neural population activity as reasonably well approximated by the extreme value distribution, and thus to conclude that a simple winner-take-all procedure that could be implemented in real neural circuits can approximate probability matching, if the weights and biases have the right values, and can also approximate all policies between maximizing and pure guessing depending on the level of the noise^7^.

### The logistic function

We now consider a variant of the scenario described above, in which we have just two mutually exclusive hypotheses. In this case it is possible to use bias terms and weights that allow us to calculate the posterior probability of one of the two hypotheses more directly, using the logistic function—the function we mentioned above that is very frequently used in setting the activations of units in neural network models. The approach is very natural when *h*_1_ corresponds to the hypothesis that some proposition is true, and *h*_2_ corresponds to the proposition that it is false, but can be applied to any situation in which there are two mutually exclusive and exhaustive alternatives. We will present the logistic function by deriving it from the softmax function for the special case of two alternative hypotheses.

We consider the calculation of the posterior probability of *h*_1_, noting that the posterior probability of *h*_2_ must be 1 minus this quantity. Specializing the softmax function of this case, we can write:

$$\rho_{1}=\frac{e^{net_{1}}}{e^{net_{1}}+e^{net_{2}}}$$

where *net*_1_ and *net*_2_ are based on the values of the biases and weights as described above. Dividing the numerator by *e*^*net*_2_^, recalling that *e*^*a*^/*e*^*b*^ = *e*^*a* − *b*^ and noting that *e*^*net*_2_^/*e*^*net*_2_^ = 1 we obtain:

$$\rho_{1}=\frac{e^{net_{1}-net_{2}}}{e^{net_{1}-net_{2}}+1}$$

Rather than compute each net input term separately and then subtract them, we can instead compute a single net input using biases and weights corresponding to the difference between the corresponding terms in each of these two expressions. That is, we define the combined net input as:

$$net=b+\sum_{j}a_{j}w_{j}$$

where *b* = *b*_1_ − *b*_2_ and *w*_*j*_ = *w*_1*j*_ − *w*_2*j*_. Replacing the bias and weight terms with their probabilistic values we have *b* = log(*p*(*h*_1_)) − log(*p*(*h*_2_)) and *w*_*j*_ = log(*p*(*e*_*j*_|*h*_1_)) − log(*p*(*e*_*j*_|*h*_2_)), and recalling that log(*a*) − log(*b*) = log(a/b), we see that if the old biases and weights corresponded to the appropriate Bayesian quantities, the new combined bias term will be equal to log(*p*(*h*_1_)/*p*(*h*_2_)) and each new combined weight *w*_*j*_ will be equal to log(*p*(*e*_*j*_|*h*_1_)/*p*(*e*_*j*_|*h*_2_)).

In terms of a single hypothesis *h* that is either true or false, the bias term becomes $\log(p(h)/p(\bar{h}))$ or log(*p*(*h*)/(1 − *p*(*h*)) and the *w*_*j*_ becomes $\log(p(e_{j}|h)/p(e_{j}|\bar{h}))$. These are quantities often used in discussions of probabilities. The first is called the *log-odds*. The second is the log of the likelihood ratio, although in this case it is the element-specific likelihood ratio, specifying the log of the ratio of the likelihood of a specific element of the evidence when *h* is true to the likelihood of that same element of the evidence when *h* is false. The overall log likelihood ratio given *n* conditionally independent elements of evidence is the sum of these quantities over all of the conditionally independent elements of the evidence.

From this we now can see that the posterior probability that some hypothesis *h* is true can be expressed as:

$$\rho =\frac{e^{net}}{e^{net}+1}$$

where the net input is the sum of a bias term equal to the log of the prior odds and each weight in the contribution from each element of the evidence is equal to the element-specific log likelihood ratio. This expression does not look exactly like the logistic function as usually written, but it is equivalent to it. We can produce the usual form of the logistic function by dividing the numerator and the denominator by *e*^*net*^, relying on the fact that 1/*e*^*x*^ = *e*^−*x*^:

$$\rho =\frac{1}{1+e^{-net}}$$

This form of the function is used in simulators since it involves calling the exp() function only once, but they are both essentially the same function.

To summarize this section: The softmax function can compute according to Bayes' formula using biases and weights corresponding to the logs of key Bayesian quantities, while the logistic function computes according to Bayes' formula using biases and weights corresponding to logs of ratios of these quantities. The minus sign in the exponentiation in the logistic function reflects a simplification of the formula that slightly obscures the relationship to Bayes' formula but makes calculation quicker. It is also worth reiterating that the softmax and logistic functions could be used to describe the outcome of a process in which one simply chooses the alternative with the largest net input, subject to Gaussian noise. In such a case we might think of the system as attempting to maximize, but appearing to be doing something more like probability matching, because the noise sometimes makes the wrong alternative come out ahead.

### Logistic additivity

Here we discuss a characteristic of patterns of data we will call *logistic additivity*. This is a condition on the relationship between the posterior probability that some binary hypothesis *h* is true, as we manipulate two independent sources of evidence, under the assumption that the sources of evidence are conditionally independent given *h* and given $\bar{h}$. It is also, at the same time, a condition on the expected output of the logistic function, given that each source of evidence has an additive effect on the net input variable that is the input to this function. Logistic additivity is of special interest for us because [as pointed out by Massaro (1989)], the original IA model failed to exhibit this pattern, thereby failing to correspond to a proper Bayesian computation and to patterns often seen in behavioral data at the same time.

We will say that logistic additivity holds for the effects of two independent sources of evidence on the probability of some outcome when they have additive influences on the **logit** of the probability of the outcome given the two sources of evidence. The logit of a probability *p* is defined as follows:

logit(p)=log(p/(1−p))

With this expression defined, we can write the statement of the condition under which logistic additivity holds as:

$$\text{logit}(p(h_{1}|e_{1},\;e_{2}))=b+f_{1}(e_{1})+f_{2}(e_{2})$$

**Figure 5:**
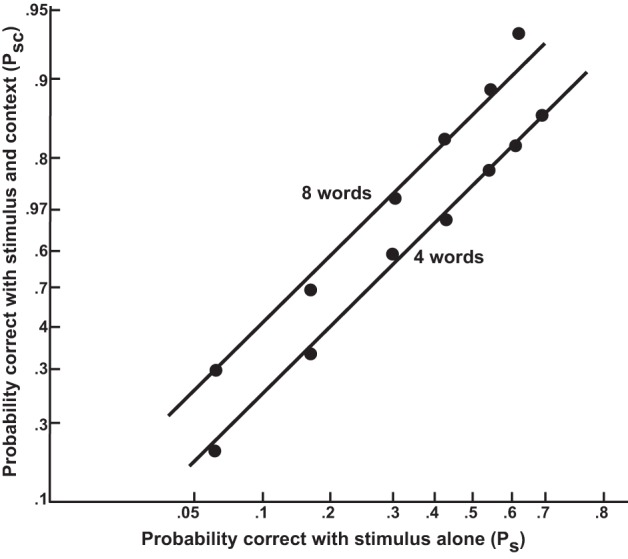
**The joint effect of context and stimulus information on probability of identifying a word correctly, displayed on axes where points are spaced according to the logit of the indicated probabilities.** The *x* axis corresponds to the logit of the probability of identifying a target word when presented without context; in the experiment (Tulving et al., 1964), this probability was manipulated by using different exposure durations ranging from 0 to 120 ms. Two curves are plotted, one for cases in which an eight-word context was provided (e.g., for the target *raspberries*: “We all like jam made from strawberries and”), and one for the case in which only the last four words of the context was provided. The curves show that the context and stimulus information have additive effects on the logit of the probability of identifying the stimulus correctly. From Morton (1969). Reprinted with permission.

This result is nice for visualization purposes since it says that for a factorial combination of different levels of *e*_1_ and *e*_2_, we should obtain parallel curves. While we will not develop this point further here, these parallel curves can be turned into parallel straight lines by appropriate spacing of points along the x axis. In his excellent early analysis of context effects in word recognition (Morton, 1969) used this approach. Further details are presented in Figure 5 and the corresponding caption.

We now show how logistic additivity follows from Bayes formula for the case of two sources of evidence *e*_1_ and *e*_2_ for hypotheses *h* and $\bar{h}$. We work from Bayes formula, using *S* = *p*(*h*)*p*(*e*_1_|*h*)*p*(*e*_2_|*h*) to represent the support for *h* and $\bar{h}$ to represent the support for $\bar{h}$, so that:

$$p(h|e_{1},\;e_{2})=\frac{S}{S+\bar{S}}$$

Dividing the numerator and denominator of this expression by $\bar{S}$:

$$p(h|e_{1},\;e_{2})=\frac{\left(S/\bar{S}\right)}{1+(S/\bar{S})}$$

It follows from this that:

$$1-p(h|e_{1},\;e_{2})=\frac{1}{1+(S/\bar{S})}.$$

If you do not see this immediately, add the two quantities together—clearly they sum to 1. Dividing the first expression by the second, we obtain:

$$p(h|e_{1},\;e_{2})/[1-p(h|e_{1},\;e_{2})]=S/\bar{S}$$

Replacing *S* and $\bar{S}$ with the products they each stand for, and taking logs of both sides, we obtain:

$$\text{logit}(p(h|e_{1},\;e_{2}))=\log\frac{p(h)}{p(\bar{h})}\;+\log\frac{p(e_{1}|h)}{p(e_{1}|\bar{h})}+\log\frac{p(e_{2}|h)}{p(e_{2}|\bar{h})}$$

The right-hand side of this equation exhibits logistic additivity, with $\log(p(h)/p(\bar{h}))$ corresponding to *b*, $\log(p(e_{1}|h)/p(e_{1}|\bar{h}))$ corresponding to *f*_1_(*e*_1_), and $\log(p(e_{2}|h)/p(e_{2}|\bar{h}))$ corresponding to *f*_2_(*e*_2_).

Working directly from the logistic function we can proceed in a similar vein to arrive at the formula expressing logistic additivity. Given that $\rho =\frac{e^{net}}{e^{net}+1}$ it follows that $1-\rho =\frac{1}{e^{net}+1}$. From these observations, it follows that ρ/(1 − ρ) = *e*^*net*^, since the denominators cancel. Taking logs of both sides and replacing *net* with its definition we have:

$$\text{logit}(\rho )=b+a_{1}w_{1}+a_{2}w_{2}$$

The idea that different sources of evidence—and in particular stimulus and context information—should exhibit logistic additivity was referred to as the *Morton*–*Massaro Law* by Movellan and McClelland (2001), and is a consequence of the assumptions of both Morton's and Massaro's (e.g., Massaro, 1989) models of how different sources of information are combined. Though neither model was explicitly formulated in Bayesian terms, it should be clear that these models follow from Bayes' formula and from the assumption that context and stimulus information are conditionally independent sources of evidence about the identity of an item in context.

Given the above analysis we can think of the logit transform of a probability (a number between 0 and 1) as converting the probability into an unbounded real number whose value exhibits additive influences arising from logs of prior odds and logs of the ratios of likelihoods of conditionally independent elements of evidence. The transform is the inverse of the logistic function, uncovering the underlying additivity of the contributions of the inputs to the function.

## Probabilistic computations in the multinomial interactive activation model

With the above background, we are finally ready to apply the ideas we have explored so far to the MIA model (Khaitan and McClelland, 2010; Mirman et al., in press). The goal of perception, according to this model, is to infer the underlying state of the world that gave rise to observed features. In this case, the goal is to infer the identity of the word and of the four letters that generated the features that reach the input to the model in a trial of a perception experiment using displays containing features in four letter positions.

**Figure 6:**
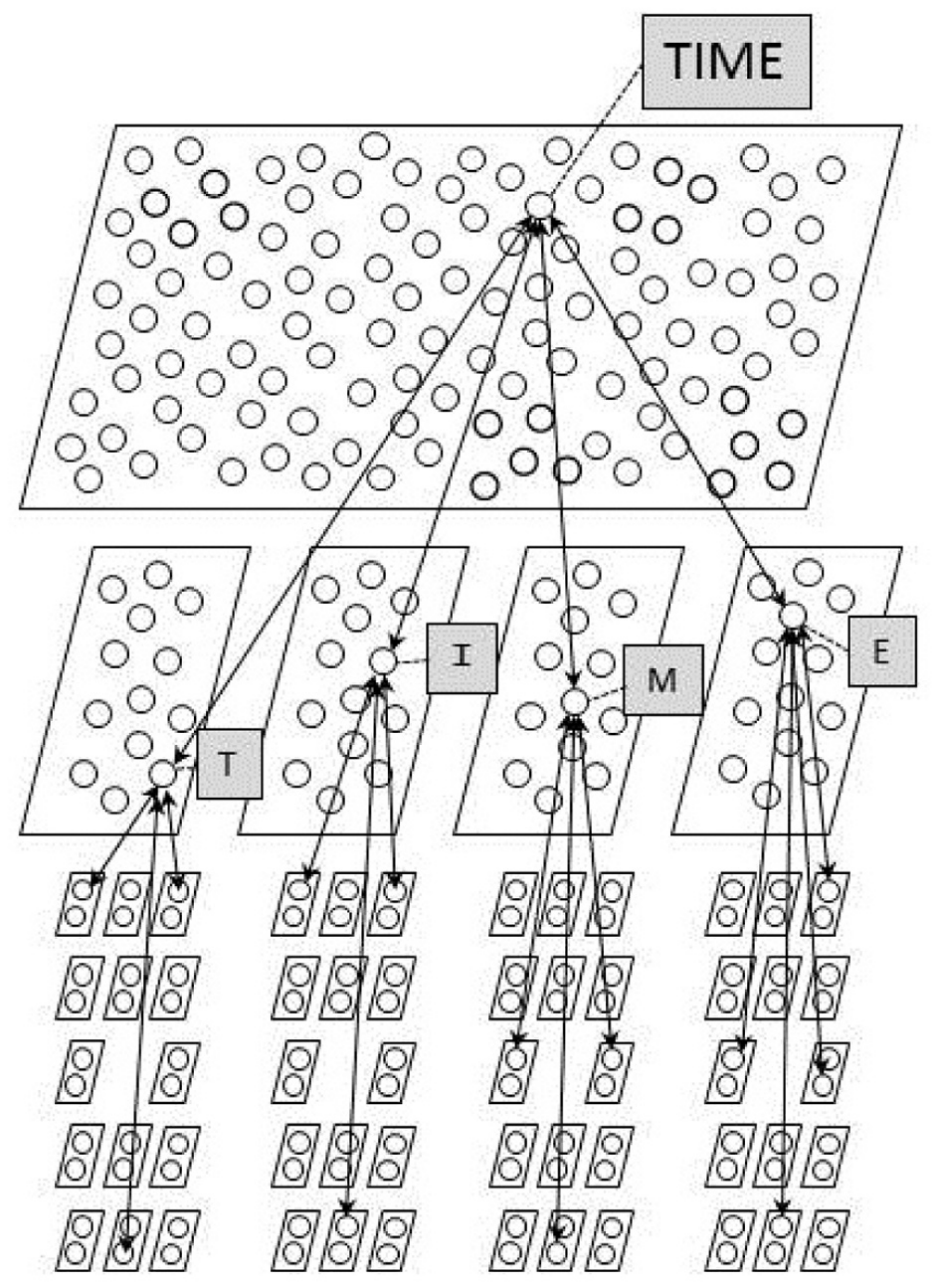
**The architecture of the multinomial interactive activation model.** Each parallelogram in the figure corresponds to a pool of mutually exclusive units, corresponding to a multinomial random variable in the probabilistic conception of the model. The softmax function is used to calculate estimates of posterior probabilities for the word units and for each pool of letter units.

A diagram of the model is presented in Figure 6. The diagram shows some of the units and a small subset of the connections in the neural network model, or equivalently, it depicts the set of multinomial random variables used in the model, and some of the constraints that influence the probability that these variables will take on particular values. The identity of the word is treated as the value of a multinomial random variable that can take on one of *n*_*w*_ values where *n*_*w*_ corresponds to the number of known words, and each word unit in the neural network model corresponds to one of the possible values this multinomial random value might take. Similarly, the identity of the letter in each position is treated as the value of one of four additional multinomial random variables each of which can take on one of 26 values corresponding to the letters of the alphabet, and each letter unit in each position corresponds to one of the values the variable for that position might take. Finally, the observed value of each feature in a given position is treated as the value of one of 14 multinomial random variables, each of which can take on either of two values (*present*, *absent*); in the neural network model, there is a separate unit for each of these two possible values within each of these 14 variables. There is a separate set of 14 multinomial variables for each position, or equivalently, a separate set of 14 × 2 feature units for each position.

Restating the goal of perception in terms of these variables, it is to infer values of the word and letter variables based on inputs specifying values of the feature variables. Note that the correct values of these variables cannot be determined with certainty, since the generative process that produces the observed features is assumed to be probabilistic. The MIA model assumes that perception produces as its outcome a sample of a possible underlying state of the world that could have generated the observed features. This sample takes the form of a set of specific values for the multinomial word variable and the four letter variables (e.g., [WORD = TIME; LETTERS = {T,I,M,E}]), and corresponds to one word unit being active and one letter unit being active in each of the four letter positions. Alternative possible underlying states are sampled probabilistically, such that the probability of sampling each possible underlying state corresponds to the actual posterior probability that this was the underlying state that generated the observed features, according to the generative model embodied in its architecture and its connection weights. The model also provides a mechanism for doing so, based on a procedure we will describe below. Before we turn to the model, however, we must establish what the posterior probabilities of different underlying states of the world are, given that we have observed a set of feature values in each position as our evidence. To do this, we must first describe the generative model assumed to give rise to observed feature arrays.

### The generative model of the multinomial interactive activation model

The generative model of the MIA model is a characterization of the process that produces the set of input features received by a participant in a letter and word perception experiment. The generative process can be envisioned with the help of Figure 6, with the proviso that the generative process runs strictly top down, whereas constraints among units run in both directions.

The first step in the generative process is to select a word at random from a list of words that are four letters long, in accordance with a base rate for each word (represented *p*(*w*_*i*_)). We then generate letters independently in each position with probability *p*(*l*_*j*_*p*__|*w*_*i*_), where we use *l*_*j*_*p*__ to represent letter *j* in position *p*^8^. Given the above procedure, the probability of a particular word *w*_*i*_ and four letters {*l*_*j*_*p*__} is:

$$p(w_{i},\;\{l_{j_{p}}\})=p(w_{i})\prod_{p}p(l_{j_{p}}|w_{i}).$$

Now using the letter sampled in each position independently, we sample values for features for each letter. As noted above, we treat the set of features as consisting of 14 separate feature dimensions, for each of which there are two explicitly represented possibilities, one that the feature is present and one that it is absent. Independently for each dimension, we select the value for a given feature dimension with probability *p*(*f*_*v*_*dp*__|*l*_*j*_*p*__)^9^.

The generative process has produced a word, a letter in each position, and a value for each feature of each letter. We will call this set of elements a *path P*_*i*, {*j*_*p*_}, {*v*_*dp*_}_ of the generative process, and subscript it with the indices of all of the selected elements, one for the word (*i*), a set of four indices {*j*_*p*_} for the letters, where *p* runs over the four positions, and the set of 4 × 14 indices {*v*_*dp*_} each specifying the value *v* (*present*, *absent*) of each feature dimension *d* of each position *p*. The probability of a given path is:

$$p(P_{i,\;\{j_{p}\},\;\{v_{dp}\}})=p(w_{i})\prod_{p}p(l_{j_{p}}|w_{i})\prod_{d}p(f_{v_{dp}}|l_{j_{p}}).$$

Simplify the notation slightly, using *p*({*v*_*d*_*p*__}|*l*_*j*_*p*__)^10^ to represent ∏_*d*_*p*(*f*_*v*_*dp*__|*l*_*j*_*p*__), this becomes:

(7) $$\begin{array}{l} p(P_{i,\;\{j_{p}\},\;\{v_{dp}\}})=p(w_{i})\prod_{p}p(l_{j_{p}}|w_{i})p(\{v_{d_{p}}\}|l_{j_{p}}). \end{array}$$

We will refer to this equation later as the *path probability equation*.

We can now consider the posterior probability of a particular combination of unobserved word and letter variables, given an observed set of features, representing this with the expression *p*(*w*_*i*_,{*l*_*j*_*p*__}|{*v*_*dp*_}). This is just the path probability of the full path involving the given word, letters, and observed features, divided by the sum of the path probabilities of all of the paths that could have generated the observed features:

$$p(w_{i},\;\{l_{j_{p}}\}|\{v_{dp}\})=\frac{p(P_{i,\;\{j_{p}\},\;\{v_{dp}\}})}{Z_{\left\{v_{dp}\right\}}}.$$

The denominator represents a quantity called the *partition function*. It stands for the sum over all *n*_*w*_ × 26^4^ path probabilities. The above equation is nothing more than an application of Bayes formula, but in a situation where the alternative hypotheses are the alternative *combinations* of possible word and letter identities that could have produced the given evidence, or ensemble of features.

Let us now consider how we could calculate the posterior probability that the word responsible for a given path was word *i*, given that we observed the set of features {*v*_*dp*_}. This will be the sum, over all paths that can generate these features starting from the word *i*, of the probabilities of these paths, divided by the sum over all of the paths that could have generated the observed features:

$$p(w_{i}|\{v_{dp}\})=\frac{\sum_{j_{1},j_{2},j_{3},j_{4}}p(w_{i})\prod_{p}p(l_{j_{p}}|w_{i})p(\{v_{d_{p}}\}|l_{j_{p}})}{Z_{\left\{v_{dp}\right\}}}$$

The summation in the numerator is the sum over the 26^4^ possible combinations of the 26 possible letters, one in each of the four letter positions, and *Z*_{*v*_*dp*_}_ is the partition function as above.

It is useful at this point to introduce the conceptual and terminological distinction between the *joint* posterior probability of a *combination* of variables and the *marginal* posterior probability of a *single* variable. The quantity *p*(*w*_*i*_,{*l*_*j*_*p*__}|{*v*_*dp*_}) is an example of a joint posterior probability (in this case, of the combination of the indexed word and the four indexed letters), whereas *p*(*w*_*i*_|{*v*_*dp*_}) is an example of a marginal posterior probability (in this case, of just the indexed word). There are also marginal posterior probabilities associated with each of the indexed letters, e.g., for the first position *p*(*l*_*j*_1__|{*v*_*vp*_}). The marginal posterior probability that a single variable has a given value is the sum of the joint posterior over all of the combinations of variables in which the variable has the given value. For example, the marginal posterior probability of word *i* is the sum over all of the combinations involving word *i* of the joint posterior probability of the combination. As we will see, some procedures naturally calculate marginal posterior probabilities, while other procedures naturally sample from joint posterior probabilities. We will consider these concepts further as we proceed.

It will simplify further analysis to note that *p*(*w*_*i*_) is a constant that can be pulled out of the summation in the numerator above, and that we can use the distributive law^11^ to rewrite ∑_*j*_1_,*j*_2_,*j*_3_,*j*_4__ ∏_*p*_ *x*_*j*_*p*__ as ∏_*p*_ ∑_*j*_ *x*_*j*_*p*__. Using these two facts, the above reduces to:^12^

$$p(w_{i}|\{v_{dp}\})=\frac{p(w_{i})\prod_{p}\sum_{j_{p}}p(l_{j_{p}}|w_{i})p(\{v_{d_{p}}\}|l_{j_{p}})}{Z_{\left\{v_{dp}\right\}}}$$

The value we obtain for each word *i* corresponds to the marginal posterior probability of the word given the observed features.

Now, let us turn to the problem of calculating the marginal posterior probability that the letter in some arbitrary letter position is letter *j*, given the full set of feature values {*v*_*dp*_} over the four positions. This probability is just the sum of probabilities of all of the paths that involve letter *j* in position *p* and the given feature values in all four positions, divided by the sum of the probabilities of all of the paths that could have generated the given features. The expression below represents this summation. We focus on position 1 to simplify the notation—analogous expressions can be written replacing the index 1 with the index of any of the other letter positions.

$$p(l_{j_{1}}|\{v_{dp}\})=\frac{\begin{array}{l} \sum_{i}\sum_{\left\{j_{2},j_{3},j_{4}\right\}}p(w_{i})p(l_{j_{1}}|w_{i})p(\{v_{d_{1}}\}|l_{j_{1}})\prod_{p\;\neq \;1}p(l_{j_{p}}|w_{i})p(\{v_{d_{p}}\}|l_{j_{p}}) \end{array}}{\begin{array}{l} \sum_{{j^{\prime }}_{1}}\sum_{i}\sum_{\left\{j_{2},j_{3},j_{4}\right\}}p(w_{i})p(l_{{j^{\prime }}_{1}}|w_{i})p(\{v_{d_{1}}\}|l_{{j^{\prime }}_{1}})\prod_{p\;\neq \;1}p(l_{j_{p}}|w_{i})p(\{v_{d_{p}}\}|l_{j_{p}}) \end{array}}$$

The expression looks complex^13^, but if we approach it slowly it should make sense. Starting with the numerator, we start with the notation for the summation over all of the *n*_*w*_ × *n*^3^_*l*_ possible paths that could have generated the given features and that involve letter *j* in position 1. The probability of each of these paths is then the product of the prior probability for the word involved in the specific path, *p*(*w*_*i*_), times a corresponding expression for each of the letters involved in the path.

Once again we can simplify. Looking first at the numerator, we can pull out the expression *p*({*v*_*d*_1__}|*l*_*j*_1__) from the summation over words and letter combinations, since this expression is constant with respect to these. Likewise, we can pull *p*(*w*_*i*_) *p*(*l*_*j*_1__|*w*_*i*_) out of the summation over letter combinations, since it too is constant in all of these combinations. We can then use the distributive law to replace ∑_{*j*_2_,*j*_3_,*j*_4_}_ ∏_*p* ≠ 1_*p*(*l*_*j*_*p*__|*w*_*i*_) *p*({*v*_*d*_*p*__}|*l*_*j*_*p*__) with ∏_*p* ≠ 1_ ∑_*j*_*p*__*p*(*l*_*j*_*p*__|*w*_*i*_) *p*({*v*_*d*_*p*__}|*l*_*j*_*p*__). In the denominator, we have partitioned the sum of the full set of path probabilities into subsets, one for each set of paths involving a different letter in position 1. We can apply the simplifications just described for the numerator to each such term in the denominator to obtain:

$$p(l_{j_{1}}|\{v_{dp}\})=\frac{p(\{v_{d_{1}}\}|l_{j_{1}})\sum_{i}p(w_{i})p(l_{j_{1}}|w_{i})\prod_{p\;\neq \;1}\sum_{j_{p}}p(l_{j_{p}}|w_{i})p(\{v_{d_{p}}\}|l_{j_{p}})}{\sum_{{j^{\prime }}_{1}}p(\{v_{d_{1}}\}|l_{{j^{\prime }}_{1}})\sum_{i}p(w_{i})p(l_{{j^{\prime }}_{1}}|w_{i})\prod_{p\;\neq \;1}\sum_{j_{p}}p(l_{j_{p}}|w_{i})p(\{v_{d_{p}}\}|l_{j_{p}})}$$

The leftmost factor in the numerator *p*({*v*_*d*_1__}|*l*_*j*_1__) now corresponds to the standard Bayesian quantity *p*({*e*}|*h*), where {*e*} is the bottom-up evidence for the ensemble of features in position 1 and *h* is the hypothesis that the letter in position 1 is letter *j*. Everything else in the numerator specifies what we will call *p*(*l*_*j*_1__|*c*), the probability of letter *j* in position 1, given the context *c*, where the context is the set of features in all of the other letter positions. Thus, we could rewrite the numerator as *p*({*v*_*d*_1__}|*l*_*j*_1__) *p*(*l*_*j*_1__|*c*). The denominator consists of a sum over all of the letters of corresponding quantities, so we can rewrite the above to express the posterior letter probability:

(8) $$\begin{array}{l} p(l_{j_{1}}|\{v_{dp}\})=\frac{p(\{v_{d_{1}}\}|l_{j_{1}})p(l_{j_{1}}|c)}{\sum_{{j^{\prime }}_{1}}p(\{v_{d_{1}}\}|l_{{j^{\prime }}_{1}})p(l_{{j^{\prime }}_{1}}|c)} \end{array}$$

This equation once again looks like Bayes' formula, but this time, we use the probability of the item given the context in place of the prior or base rate. This should make sense: We can think of what we are doing here as using the context to set the “prior” probabilities of different letters to context-specific values, combining these context specific prior probabilities with the contribution of the evidence, to calculate the total support for each of the possible alternative letters.

### Alternative procedures for calculating and sampling posterior probabilities given observed feature arrays

The above can be thought of as a mathematical characterization of the true posterior joint and marginal probabilities of each word and of each letter in each position, conditional on observing some set of features {*v*_*dp*_}, under the generative model. How might we calculate, or sample from, these quantities during perception?

We now describe two different ways to calculate the *marginal* posterior probabilities of words and letters—a *undirectional* method and an *interactive* method. After that we will describe how the updating procedure used in the MIA model allows us to sample from the *joint* posterior distribution, and (as a byproduct) also the marginal posterior distribution over words and over letters in each position.

#### A unidirectional calculation method

**Figure 7:**
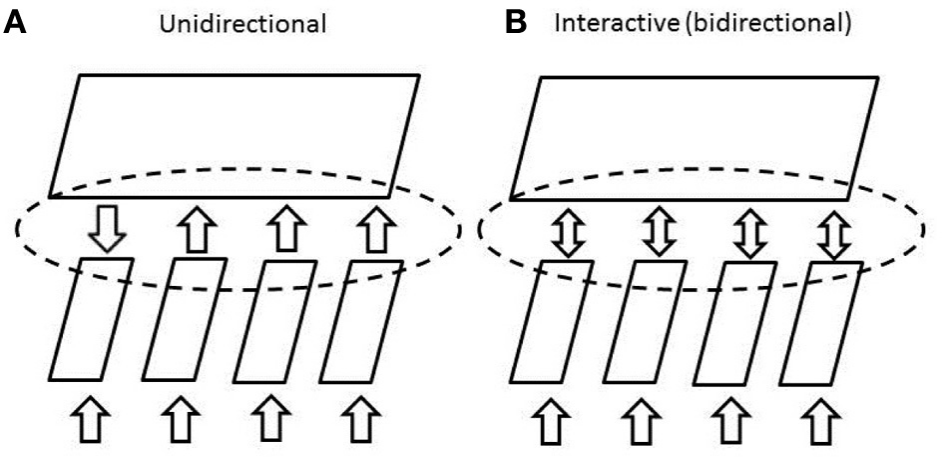
**Schematic diagram of flow of computation for: (A) unidirectional procedure for calculating the posterior probabilities of letters in the first position and (B) bi-directional procedure for calculating the posterior probabilities of all four letters.** The dashed ovals highlight the differences between the two procedures. In the unidirectional procedure, calculation proceeds upwards to the word level from positions 2, 3, and 4, and downward only for position 1. In the interactive procedure, calculation proceeds upwards and downwards in all four positions.

Our first calculation method is completely non-interactive—information flows in a single direction between each pair of pools, as shown in Figure 7A^14^. Both Figure 7A and the text below apply to the particular case of calculating the posterior probability of possible letters in position 1, given the full set of features {*v*_*dp*_}.

1. For each letter in each position, including position 1, we first calculate *p*({*v*_*d*_*p*__}|*l*_*j*_*p*__). This corresponds to the *upward* arrows from each feature array to each letter array in Figure 7A.
2. For each word, we then calculate *p*(*w*_*i*_) ∏_*p* ≠ 1_ ∑_*j*_*p*__*p*(*l*_*j*_*p*__|*w*_*i*_) *p*({*v*_*d*_*p*__}|*l*_*j*_*p*__). This is the support for each word, given the feature information in all positions other than position 1, and we will thus call it *S*_*i*/1_^15^. This corresponds to the three *upward* arrows from the position 2, 3, and 4 letter arrays to the word array in Figure 7A.
3. For each letter *j* in position 1, multiply each of the above word-specific terms by *p*(*l*_*j*_1__|*w*_*i*_) and sum over words to obtain: ∑_*i*_*p*(*l*_*j*_1__|*w*_*i*_)*S*_*i*/1_. These quantities are the *p*(*l*_*j*_1__|*c*) terms we need, and the computation corresponds to the downward arrow in Figure 7A from the word level to the letters in position 1.
4. Finally, calculate *p*(*l*_*j*_1__|{*v*_*dp*_}) using the posterior letter probability equation (Equation 8), taking *p*({*v*_*d*_1__}|*l*_*j*_1__) from step 1 and the *p*(*l*_*j*_1__|*c*) from step 3.

The procedure can, of course, be applied to any letter position, just exchanging the roles of position 1 and any other position.

***A drawback of the unidirectional method***. The method just reviewed is basically consistent with the ideas of Massaro (1989) and Norris and McQueen (2008) and elsewhere. Both argue that when identifying the letter (or phoneme) in a particular string position, we must separately calculate context and stimulus support for each alternative, then combine these quantities only as the final step of our computation. The idea seems sensible when we think about using preceding context to help recognize the next phoneme in a spoken word. We can imagine generating expectations based on the input received so far for the phoneme next to come, then combining these expectations with bottom-up information about this next phoneme to compute its posterior, iterating this procedure for each successive phoneme. However, experimental evidence (e.g., Rumelhart and McClelland, 1982) supports the view that perception of letters in every position of a briefly-presented word benefits from contextual influences from all other positions. The data indicates that the perception of each letter should benefit from the context provided by all of the other letters, and that these computations should be carried out in parallel, so that these influences can occur while the input is available for identification. Subsequent context also affects phoneme perception from spoken input, even though the context does not arrive until after the target phoneme (Warren and Sherman, 1974; Ganong, 1980), a key finding motivating the interactive architecture of the TRACE model of speech perception (McClelland and Elman, 1986).

In general, to maximize the use of context, it seems desirable to calculate posterior probabilities for each letter using the context provided by all the other letters, and it could be useful to calculate posterior probabilities for words, based on all of the letters, as well. The original IA model achieved something close to this, but not exactly—many of the complaints by Massaro and later by Norris et al. (2000; Norris and McQueen, 2008) focused on the fact that the model did not get the posterior probabilities exactly right; indeed, as documented by McClelland (1991) and Movellan and McClelland (2001), the original IA model failed to exhibit logistic additivity. Here we consider how the correct posterior probabilities can be calculated by an interactive procedure.

To calculate the posterior probabilities over words, we should of course include input from all four positions in the corresponding calculation of the *S*_*i*_ for each word. To calculate the context terms for a given position—say position 1—we have to exclude its contribution to the word level to obtain the appropriate *S*_*i*/1_ values. It would be possible to calculate *S*_*i*_ along with *S*_*i*/1_, *S*_*i*/2_, *etc*., separately in parallel, but it seems redundant and computationally wasteful. Fortunately, there is a simple way to avoid the redundancy.

#### A parallel, interactive method

The approach we now consider is a specific instance of the approach proposed by Pearl (1982)—it is not the procedure we use in the MIA model, but it is useful to understand both procedures, and the differences between them. Pearl's approach allows processing to occur in parallel for all four letter positions, relying on the bi-directional propagation of information, as shown in Figure 7B, minimizing the redundancy just noted. The key observation (specialized for our specific circumstances) is that the posterior probability for each word contains a product of terms, one from each letter position. The term from a given letter position *p* to each word unit *i* is ∑_*j*_*p*(*l*_*j*_*p*__|*w*_*i*_) *p*({*v*_*d*_*p*__}|*l*_*j*_*p*__). Suppose we call each of these quantities *r*_*i*_*p*__. Then we can calculate the full bottom-up support for each word combining the *r*_*i*_*p*__ across all four positions, saving the *r*_*i*_*p*__ values so that we can divide them back out in calculating the *p*(*l*_*j*_*p*__|*c*) factors for each position. In more detail, here is the procedure:

1. For each letter in each position, calculate *p*({*v*_*d*_*p*__}|*l*_*j*_*p*__).
2. For each word, then calculate *S*_*i*_ = *p*(*w*_*i*_) ∏_*p*_*r*_*i*_*p*__ where *r*_*i*_*p*__ is as defined above, using the values calculated in step 1. *S*_*i*_ represents the total support for each word, given the feature information in all positions and the prior word probability, and can be used to calculate the posterior probability of each word by dividing through by the sum of all of the *S*_*i*_.
3. For each letter position, we now calculate the appropriate top-down term by dividing *S*_*i*_ by *r*_*i*_*p*__ to obtain *S*_*i*/*p*_. We then proceed to calculate, for each letter *j*, *p*(*l*_*j*_*p*__|*c*) = ∑_*i*_*p*(*l*_*j*_*p*__|*w*_*i*_)*S*_*i*/*p*_ as in the unidirectional procedure.
4. For each position, we finally calculate *p*(*l*_*j*_*p*__|{*v*_*dp*_}), using the *p*({*v*_*d*_*p*__}|*l*_*j*_*p*__) from step 1 and the *p*(*l*_*j*_*p*__|*c*) from step 3.

This procedure is a specific instance of the one proposed by Pearl (1982) for unidirectional causal graphical models (models in which causality propagates only in one direction, as in our generative model), subject to the constraint that each multinomial variable (i.e., each a set of mutually exclusive hypotheses) in the graph has at most one parent, i.e., one variable that it is conditioned on in the generative model. The generative model underlying the MIA model is an example of such a graph: In the generative model, the multinomial word variable has no parents; each of the four multinomial letter position variables depends only on the word variable; and each of the 14 binomial feature dimension variables in each letter position depends only on the letter variable for that position. The method allows for iterative, i.e., interactive updating; as new information arrives at any of the variables, it can be propagated through to update all of the other variables. There is an inherent sequentiality moving upward and then downward, but information can flow back and forth in both directions. If feature information built up gradually over time, the process could be iterated repeatedly, updating all of the variables as new evidence arises.

Pearl's method is an elegant and general method, and is now a long established part of the fabric of probabilistic computation. Interestingly, the idea did not come up in the development of the IA model, even though the key idea of dividing back out one's contribution to a parent when receiving top-down input from the parent was proposed by Rumelhart (1977). Perhaps one reason why Rumelhart did not suggest we explore this idea when we were developing the original IA model may be that Pearl's method requires each multinomial variable to keep separate track of its bottom-up and top-down values. What gets passed up in Pearl's algorithm is strictly feed-forward information; what gets passed down to a given multinomial variable carefully excludes the information that came up through it, and must be kept distinct^16^. A feature Rumelhart found pleasing in the original IA model was that the computation was entirely homogeneous. In an early talk on the model, he had on one of his transparencies: “activation is the only currency” transmitted between units in the network.

**Figure 8:**
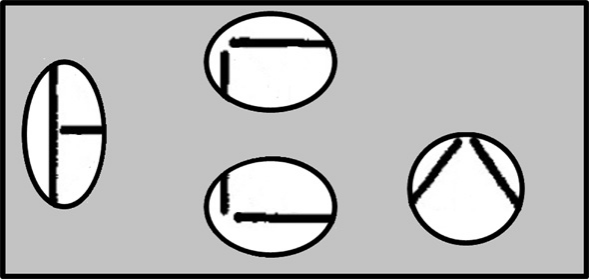
**A few features from each position of a three-letter word.** Based on the Rumelhart and Siple font, there are two consistent letters in each position, and four consistent words.

An important characteristic of Pearl's approach is that the posterior probabilities calculated for each variable are marginalized over all possible values of all of the other variables. To see what this means concretely, consider the set of features shown in Figure 8. The features in each position are consistent with two letters (H or F in the first position, E or O in the second position, and W or X in the third)^17^. The features are also consistent with four words: FEW, FOX, HEX, and HOW^18^. Pearl's algorithm will allow us to calculate that these words and letters are the most likely. Ignoring differences in word frequency, the words would all be equally likely, and so would the letters. If we selected one word from those that are equally probable, and one letter from each position from those that are equally probable, we could end up with the conclusion that the word is FEW but that the letters are H, O, and X (letters that, together, don't even form a word).

The approach used in the MIA model samples from the *joint posterior* of the generative model. That is, it samples from the space of composite hypotheses consisting of one word and one letter in each position. For example, the joint hypothesis [WORD = FEW; LETTERS = {F,E,W}] is one such hypothesis, while the hypothesis [WORD = FEW; LETTERS = {H,O,X}] is another. The first of these joint hypotheses is far more likely than the second. The MIA model assumes that perceivers select among alternative joint hypothesis weighted by their overall probability. We now turn to the procedure used for doing this.

#### Sampling from the joint posterior in the MIA model

The final approach we will consider, the one used in the MIA model, is based on the Bayesian procedure known as *Gibbs sampling*, and was used in the Boltzman machine by Hinton and Sejnowski (1983). Gibbs sampling is discussed in more detail below; for now, we note that Gibbs sampling is a procedure used to sample from the joint posterior distribution of a probabilistic model by iteratively updating the states of unobserved variables probabilistically, based on current values of observed and other unobserved variables. We present the specific version of these ideas used in the MIA model, which have been adapted and specialized for our case.

In the model, there are word, letter, and feature units as illustrated in Figure 6, and weights are considered to be bi-directional, as in the figure, but their values are defined only in one direction. At the word level, each word unit has a bias term, corresponding to the log of its prior probability, log(*p*(*w*_*i*_)). The connection weight linking each word to each letter is set equal to log(*p*(*l*_*j*_*p*__|*w*_*i*_)), and the weight linking each feature to each letter is set to log(*p*(*f*_*v*_*dp*__|*l*_*j*_*p*__)).

We specify the input to the model by setting the values of the feature units to correspond to a specific input feature array. With all units at the letter and word levels initially off, we proceed as follows:

1. For each position, we calculate each letter's net input. Since the word units are all off, there will be no contribution from the word level, and each letter unit's net input will correspond to log *p*({*v*_*d*_*p*__}|*l*_*j*_*p*__).
2. Within each letter position we then select one unit to be on, using the softmax function to calculate the probability of selecting each letter, given the net inputs to all of the letter units.
3. We then calculate the net input to each word unit, based on the single active letter unit in each position.
4. We then select one word unit to be on, again using the softmax function and the net inputs to all of the word units.
5. We calculate each letter's net input again, noting that now, one unit at the word level is active on each iteration, providing top-down input that affects the net input to each letter unit, in addition to the bottom-up input coming in from the feature level.
6. We then select one letter unit to be on in each letter position, using softmax.
7. We repeat steps 3–6 several times, then stop with one word unit active and one letter unit active in each position.

This iterative process in steps 3–6, which corresponds to Gibbs sampling, is called “settling.” The initial bottom-up pass in steps 1–2 helps the network to start the settling process from an initial state usefully constrained by the featural input.

The state of activation in the network after settling for many iterations will be a sample from the joint posterior of the generative model (we will consider why this is true below). That is, if we ran this whole procedure a very large number of times, and counted the number of times the pattern at the end of settling corresponded to each possible joint hypothesis (one word and one letter in each position), the proportion of times the network settled to each such pattern would correspond to the posterior probability of the corresponding joint hypothesis.

Running the above procedure hundreds of thousands of times would not be very efficient, but we do not propose that perception involves such a process. Instead, we propose that each trial of a perceptual experiment involves a single instance of running the above procedure. Each such instance generates a single sample from the above process, capturing the probabilistic nature of performance in behavioral experiments. In a perceptual experiment where the task is to identify the letter in a specified position (as in most of the experiments modeled using the original IA model), we can imagine that the participant simply reads out the identity of the letter corresponding to the active unit in the appropriate position. Note that this procedure is a way to use a sample from the joint posterior as a sample from the marginal posterior for a particular multinomial variable (e.g., the letter in the specified position).

***Relationship to Gibbs sampling and Boltzmann machines***. The above procedure is related to the updating procedure proposed for Boltzmann machines by Hinton and Sejnowski (1983, 1986). One difference is that in the original Boltzmann machine, units are not organized into pools corresponding to multinominal random variables. Rather, each unit is treated as a separate (binary) random variable. Units are updated one at a time, selecting the next unit to update sequentially and at random, using the logistic function. Our network is similar, but our units are organized into pools, each corresponding to a single multinomial variable, such that only one unit per pool/variable is allowed to be active at one time. In both cases, after an initial burn-in period (corresponding to what we called settling above), networks visit global states with probability proportional to *e*^*G*_*s*_/T^, where *G*_*s*_ is the goodness of the state and *T* corresponds to the temperature. The goodness of a state is defined as:

$$G(s)=\sum_{i<j}a_{i}a_{j}w_{ij}+\sum_{i}a_{i}b_{i},$$

where the summation runs over all pairs of units (with each pair of units counted only once) and *w*_*ij*_ corresponds to the bi-directional weight between the two units^19^. Additional terms can be included to represent external inputs to units, but these can be captured using weighted inputs from units whose activations are treated as clamped, as the set of input feature units are in our procedure.

For the MIA model, the goodness of a state in which one word is active and one letter in each position is active, given a set of input feature values clamped onto the input units, is given by what we will call the MIA goodness equation:

(9) $$\begin{array}{l} G(s|\{v_{dp}\})=b_{i}+\sum_{p}\left(w_{i,j_{p}}+\sum_{d}w_{j_{p},v_{d_{p}}}\right) \end{array}$$

Here *i* indexes the single word unit that is active at the word level, the four values of {*j*_*p*_} (one for each position *p*) index the active letter units in each of the four positions *p*, and the set of 4 times 14 values of {*v*_*d*_*p*__} represent the indices of the active feature-value units on each feature dimension in each feature position. The activations of the units involved are not expressed as variables because they are all equal to 1; no other terms occur in the goodness because all other units' activation values are 0.

As an exercise, the reader can check that the goodness of a state as represented by the MIA goodness equation is equal to the log of the probability of the corresponding path under the generative model, by taking the log of the path probability equation (Equation 7). Given that this is so, if we run our network at some temperature *T*, then the network will visit this state with probability proportional to *e*^log(*p*(*P*_*s*_))/*T*^, where *p*(*P*_*s*_) is the probability of the path corresponding to state *s*. The posterior probability of visiting this particular state given the particular feature values can be calculated by dividing through by the sum of the exponentiated and *T*-scaled goodnesses of all of the states that can be visited given the feature values:

$$p(S|\{v_{dp}\}/T)=\frac{e^{G_{s|\{v_{dp}\}}/T}}{\sum_{s^{\prime }}e^{G_{s^{\prime }|\{v_{dp}\}}/T}}$$

For the case where *T* is equal to 1, we obtain:

$$p(S|\{v_{dp}\})=\frac{e^{G_{s|\{v_{dp}\}}}}{\sum_{s^{\prime }}e^{G_{s^{\prime }|\{v_{dp}\}}}}$$

The probability that the network is in state *S*_*i*_ after settling is thus equal to the posterior probability of the state, given the evidence.

Hinton and Sejnowski (1983, 1986) focused on the task of finding the single best joint hypothesis using a process they called *simulated annealing*. In this process, one engages in a similar sequential update process to that described above, but with gradually reducing temperature. The procedure we have described operates at a fixed temperature. At lower temperatures, the preference for units with stronger net inputs is amplified, and as *T* goes to zero, the procedure will allocate all of the probability to the alternative with the largest net input. Gradually lowering the temperature corresponds to gradually increasing the relative probability of visiting the alternatives with the largest posterior probability. It may be worth noting that a gradual change in the clarity of evidence can have a similar effect as a gradual change in temperature, or that running the procedure when the evidence is very weak can be similar to running the procedure at very high temperature. Thus, perception with very weak stimuli may correspond approximately to running the model at very high temperature, and gradual buildup of information over time may correspond to simulated annealing. These ideas may be worth developing further in extensions of the MIA model.

***Why does the MIA model sample correctly from the posterior?*** So far we have stated without proof that “after a burn-in period” and at fixed temperature, states are sampled in proportion to *e*^*G*(*s*)/*T*^. How do we know that this is true? For particular cases, we can demonstrate the validity of this result via stochastic simulation, and we have done so for several cases, showing results for one specific case in Mirman et al. (in press). The fact that it is true for all cases follows from the fact that the sampling procedure we are using is an instance of a Gibbs sampling procedure, introduced by Geman and Geman (1984). The Gibbs sampler (named after the physicist J. W. Gibbs) is widely used to sample from posterior probability distributions in applications of Bayesian inference.

A Gibbs sampling procedure is a procedure that obtains samples from the joint posterior of a set of random variables by successively updating sampled values of individual probabilistic variables conditional on the values of other variables. Concretely in our case, we are updating the multinomial word variable based on the letter variables and each letter variable based on the word variable and the appropriate position specific feature variables. We can see our procedure as sampling from the conditional distribution of the word variable based on the values of the feature and letter variables on each update at the word level, and as sampling from the conditional distribution of each letter variable, based on the values of the word and feature variables, on each update at the letter level. After burn-in, the overall state after each update is a sample from the joint distribution over all of the variables. The statement that such states are samples from the joint posterior means that the probability of visiting each state (at equilibrium, i.e., after a burn-in period) is equal to the posterior probability of the state.

Two properties of our sampling procedure are necessary to ensure that it accurately samples from the posterior (Hastings, 1970). First, the process must be *ergodic*—it must be possible to get from any state to any other state in a finite number of steps. Taking the feature units' values to be clamped, we are concerned only with states corresponding to a joint specification of a word and four possible letters. The process is ergodic if it is possible to get from any state of the word and letter units to any other state of these units. This property holds in our case, because all of the probabilities encoded in the weights are non-zero, making it possible (a) to visit any possible state of the letter units given an active word unit and a set of active feature values, and (b) to then visit any possible state of the word units given a set of active letter values. In our case, then, it is possible in principle to get from any state to any other state in one update cycle, consisting of one letter update and one word update. So our model is ergodic^20^.

The second critical property is that the updating process exhibits *detailed balance*. A stochastic updating process is said to have detailed balance with respect to a particular probability distribution {π} = {…, π_*i*_, …, π_*j*_…}) over possible states if the probability of being in state *i* and transitioning to state *j* is equal to the probability of being in state *j* and transitioning to state *i*:

$$\pi_{i}p(i\to j)=\pi_{j}p(j\to i),$$

or equivalently,

$$\frac{p(j\to i)}{p(i\to j)}=\frac{\pi_{i}}{\pi_{j}},$$

If a stochastic updating process has this property, it will converge to the equilibrium distribution {π} in which the probabilities of states *i* and *j* are π_*i*_ and π_*j*_ respectively; ergodicity ensures that we can get to the equilibrium distribution from any starting state^21^.

Intuitively, the detailed balance condition can be seen as a way of expressing what it means for a probability distribution to be at equilibrium, or stationary. Referring to the first version of the equation, we can read it as saying that at equilibrium, the probability of being in state *i* and then transitioning to state *j* should be equal to the probability of being in state *j* and transitioning to state *i*. If this is true for all pairs of states, and if we can get from any state to any other state, then the distribution over states will stay constant as we continue to update. It is important to note that it is not the states themselves but the *distribution over states* that is stationary. On each update, the state may change, but the probability distribution over states, conceived of as the proportion of times an infinitely large ensemble of instances of the process is in each possible state, can still remain stationary. This is so because in the ensemble, the detailed balance condition stipulates that the probability of being in state *i* and transitioning to state *j* is equal to the probability of being in *j* and transitioning to *i*.

We have just seen that if we are already at equilibrium (i.e., if the ratios of probabilities of particular states are equal to the ratios of the corresponding transition probabilities) we will stay there. But what if, at a certain time, the distribution of states is not yet at equilibrium? In that case, if the transition probability ratios are equal to the equilibrium probability ratios, the transitions will tend to move the distribution toward the stationary distribution. We will not prove this statement but we will consider an example a bit later showing how movement toward the correct stationary distribution does occur.

To show that our updating procedure will sample from the posterior distribution of the MIA model, we must show that its state transitions are balanced with respect to the posterior probabilities of the paths associated with these states, i.e., that the transition probabilities between states *i* and *j* are in balance with the posterior path probabilities. To do so, it is easier to work with the second version of the statement of the detailed balance condition. Working with this version, we would like to show that the ratio of the transition probabilities between any two states is equal to the ratio of the posterior probabilities of the generative paths corresponding to these states. Designating these states and the probabilities of the corresponding paths with the subscripts *i* and *j*, this corresponds to the expression:

$$\frac{p(S_{j}\to S_{i})}{p(S_{i}\to S_{j})}=\frac{\pi_{i}}{\pi_{j}}.$$

For concreteness, let's consider a specific case. Suppose that the input features are the correct values of the features of the word TIME, and that the correct word is active at the word level, and the correct letter is active in positions 2, 3, and 4. Let state *S*_*I*_ be the state in which, in addition to the above, the letter I is active in the first position and let state *S*_*T*_ be the state in which, in addition to the above, the letter T is active in the first position, and let π_*I*_ and π_*T*_ represent the probabilities of the corresponding paths of the generative model. Using these indices, the above would then correspond to:

$$\frac{p(S_{T}\to S_{I})}{p(S_{I}\to S_{T})}=\frac{\pi_{I}}{\pi_{T}}.$$

Based on the visual similarity between I and T in the Rumelhart and Siple font, the paths associated with these states should be among the most probable, although state *I* should be less probable that state *T*. Now, suppose we are in state *I* and we are about to update the state of the first-position letter variable. We calculate the net input to each letter unit based on the active features and the active letters, and we then select the letter to activate according to the softmax function. The probability of transitioning to state *T*, i.e., of selecting *T* as the next letter, is $\frac{e^{net_{T_{1}}}}{\sum_{j}e^{net_{j_{1}}}}$, where *net*_*T*_1__, the net input to the unit for letter *T* in position 1, is:

$$\log(p(l_{T_{1}}|w_{\text{TIME}}))+\sum_{d}\log(p(f_{v_{d_{1}}}|l_{T_{1}}))$$

so that *e*^*net*_*T*_1__^ is *p*(*l*_*T*_1__|*w*_TIME_) ∏_*d*_*p*(*f*_*v*_*d*_1___|*l*_*T*_1__). Similarly, suppose we are in state *T* and we are about to update the state of the first-position letter variable. We proceed as before, and find that the probability of transitioning to state *I* is $\frac{e^{net_{I_{1}}}}{\sum_{j}e^{net_{j_{1}}}}$, where the net input to the unit for letter *I* in position 1 is:

$$\log(p(l_{I_{1}}|w_{\text{TIME}}))+\sum_{d}\log(p(f_{v_{d_{1}}}|l_{I_{1}}))$$

and *e*^*net*_*I*_1__^ is *p*(*l*_*I*_1__|*w*_TIME_) ∏_*d*_*p*(*f*_*v*_*d*_1___|*l*_*I*_1__). The ratio of these two transition probabilities, $\frac{p(S_{I}\to S_{T})}{p(S_{T}\to S_{I})}$ is then:

$$\frac{p(l_{T_{1}}|w_{\text{TIME}})\prod_{d}p(f_{v_{d_{1}}}|l_{T_{1}})}{p(l_{I_{1}}|w_{\text{TIME}})\prod_{d}p(f_{v_{d_{1}}}|l_{I_{1}})}$$

They have the same denominator, which cancels out. This ratio is the same as the ratio of the posterior probabilities of each of the two paths, since the path probabilities share all of the other factors in common as well as the same denominator, and again everything else cancels out.

It would be tedious to repeat the above analysis for all possible pairs of states that might arise in the course of our sampling process. Luckily, there was nothing special about the particular case we just considered. The same argument can be applied for any pair of states differing only by the letter that is active in one of the four letter positions, given any set of clamped features. Furthermore, an analogous argument can be applied for any two states differing only by the word that is active. Since all the transitions are from one letter in a given position to another letter, or from one word to another word, this covers all of the transitions.

This completes the proof that the MIA model exhibits detailed balance, and we previously saw that it was ergodic. It follows, then, that the model samples states with probabilities corresponding to the posterior probabilities of the corresponding paths through the generative model.

In the context of the example we were working with above, we can now observe that the distribution of states tends to move toward the correct equilibrium distribution, at least in a simple specific case. Consider, for concreteness, an ensemble of 1000 separate instances of our network, and let an arbitrary fraction be in state *I* and the rest be in state *T* just before we update the multinomial variable for the first letter position in each of these 1000 instances. As one possibility, all of the networks could be in the *I* state. Now, we note that our update procedure is unaffected by the previous active letter in the first letter position (it depends only on the state of the feature and word units—the other multinomial variables in the system). Relying on the same reasoning we worked through above, it should be clear that the update in each network will put the system in state *T* with a probability proportional to π_*T*_ = *p*(*P*_*T*_), and in state *I* with a probability proportional to π_*I*_ = *p*(*P*_*I*_), and thus the ratio of the proportion of networks in states *I* and *T* will tend toward $\frac{p(P_{T})}{p(P_{I})}$ after the update. Thus, in this case, we can move from a distribution far from equilibrium to something much closer to it in just a single update step. We have not considered what would happen in a full range of cases, but perhaps this example helps support the intuition that, in general, the distribution of states will tend to move toward the equilibrium distribution, if the transition probability ratios are in balance with the posterior path probabilities.

***A few practicalities***. It is important to be aware of two things when using Gibbs sampling and related procedures. First, it takes some time for the settling process to reach the stationary distribution. It is difficult to know how many iterations of settling to allow before taking the state of the network as a valid sample from the stationary distribution, and testing for stationarity is not easy. Second, while in principle it is possible to transition from any state to any other state, in practice adjacent states tend to be correlated, and it may take a long time to make a transition between quite different, but equally good possibilities. For example, for the display in Figure 8, the time needed to transition from the interpretation [WORD = FEW; LETTERS = {F,E,W}] to the interpretation [WORD = HEX; LETTERS = {H,E,X}] may be quite long. It may, in fact, be quicker to get a set of decent samples by restarting from a blank starting place several times. This is how we proceeded to sample from the MIA model in Mirman et al. (in press). This is appropriate for our purposes, given that we think of each trial in a perceptual experiment as corresponding to a single case of settling to a perceptual interpretation, corresponding to a single sample from the posterior.

## Summary and discussion

The analysis presented above supports the assertion that interactive processing can be consistent with principled Bayesian computation. It is hoped that the analysis will lay to rest the in-principle concern about this matter. It is true that not all versions of interactive models can accurately capture Bayesian computations, but it should now be clear that at least some can. Many questions, or course, remain. In this section I will briefly consider two issues: First, what was wrong with the original IA model? Second, can some of the assumptions made in demonstrating that the MIA model can correctly sample form the posterior of the given generative model be relaxed, and still allow for proper probabilistic computations, or a good approximation to such computations?

### What was wrong with the original IA model?

Complaints about the adequacy of the original IA model (e.g., Massaro, 1989; Norris and McQueen, 2008) have centered on the bi-directional propagation of activation signals, but in fact, the original IA model failed to produce the pattern of logistic additivity one would expect even in the absence of interactive processing: the problem arose even when two sources of bottom-up evidence were combined (McClelland, 1991). This occurred because the particular activation and response selection assumptions used in the original IA model distorted the contributions of two different sources of evidence. Specifically, the original model applied the softmax function, not to the net inputs to units, but to activations of units—activations that had already been subjected to other non-linearities. These non-linearities did not prevent the model (or the TRACE model of speech perception) from capturing qualitatively a wide range of contextual influences on perception, but did contribute to the model's failure to exhibit logistic additivity.

Even if the problem with the original IA model's activation function were corrected, however, there could still be distortions of proper probabilistic computation in a deterministic model like the original IA model, as the IA model's critics claimed. To see this, consider first the following unidirectional model, which would not produce a distortion. In this model, we use the architecture and connection weight values of the MIA model. However, we make two changes: (a) we compute unit activations in the various layers of the model, setting them to continuous values based on the softmax function rather than selecting one to have an activation of 1 and all others to have an activation of 0; and (b) we allow only a unidirectional flow of processing, as in the unidirectional procedure described previously and depicted in Figure 7A. The activations so computed will correspond exactly to the probabilistic quantities that we could have computed directly—the net inputs, which are the sums of logs of relevant probabilistic quantities will be turned back into the relevant probabilistic quantities by the exponentiation operation applied to the net input values in the softmax function. Now consider a version of this model, in which, instead of assumption (b), we allow word level activations to be computed based on letter level activations in all four letter positions, and we then send top-down signals back to the letter level from the word level based on all four letters instead of just three. This will clearly produce a distortion of the resulting activations, unless we take care (as Pearl did in his procedure) to divide back out of the top-down input to each letter position its own contribution to the activation at the word level. Based on these considerations, it appears that the original IA model may have failed to carry out proper Bayesian computations on two counts: it distorted these computations due to its basic activation assumptions and it distorted them due to its failure at lower levels to take back out its own contribution to the signals it received from higher levels.

### Relaxing some of the assumptions of the MIA model

The MIA model makes some assumptions that were helpful in the analysis presented above. Among them are (1) we allowed just one unit to be active at a time in each pool corresponding to a multinomial random variable, (2) unit activation values are restricted to the values 0 and 1, and (3) units are updated according to a strict alternation schedule. None of these features are likely to hold in real neural networks. Would it still be possible to carry out proper probabilistic computations if some or all of these assumptions were relaxed? The exact limits of the conditions under which a (real or artificial) neural network could carry out proper Bayesian computations are not fully known, and further work will be required to further our understanding of this point. For now, I offer the conjecture that perhaps all of these assumptions can be relaxed, based on the following considerations.

First, in McClelland (1991), I presented simulations showing that logistic additivity of factors affecting stimulus and contextual influences on letter identification could be observed in three different variants of the original IA model, whereas the original model violated logistic additivity. Since logistic additivity of stimulus and context effects should be observed under the generative model underlying the MIA model, these findings are consistent with the conjecture above.

One of the three variants I considered in McClelland (1991) was a Boltzmann machine version of the original model. This variant is very similar to the MIA model, with these differences: (a) units within a pool are mutually inhibitory (there are negative connections between them) but they were not strictly mutually exclusive as in the MIA model and (b) unit activations were updated completely at random, as in the standard implementation of a Boltzmann machine. A mathematical analysis presented in McClelland (1991) demonstrated that logistic additivity follows from the assumptions of this model, and in McClelland (1998) I extended this analysis by showing that if the weights and bias terms in this variant of the model are set to the logs of the same probabilistic quantities used in the MIA model, then after settling to equilibrium at a temperature of 1, the relative probabilities of states with exactly one active word unit and exactly one active letter unit in each position would correspond to the relative posterior probabilities of the corresponding paths from the generative model.

The Boltzmann version of the IA model just considered still makes use of binary units. Could samples from the posterior still be obtained in models using continuous activation values for units in the neural network? It seems likely. The other two variants of the original IA model that exhibited logistic additivity in McClelland (1991) did use continuous activation values—in fact, these variants also retained the activation assumptions used in the original IA model. What differentiated these variants from the original model were the assumptions about sources of variability. In the original model, processing was completely deterministic and variability only affected response choices based on activations calculated deterministically, whereas in the two variants considered in McClelland (1991), variability was present either in the external inputs to the model or in the calculation of the net input to each of the units in the network. In both variants, the response choice after a period of settling was determined by selecting the most active unit within a mutually exclusive pool of units (e.g., the units for letters in one of the four letter positions). Yet another variant that used continuous activation values that also exhibits logistic additivity was presented in Movellan and McClelland (2001). These demonstrations of logistic additivity are largely based on simulations; proving that these variants produce logistic additivity is challenging, although some analysis under certain limiting conditions was provided for the third variant in Movellan and McClelland (2001). These findings are consistent with the conjecture that interactive networks that incorporate variability either in their inputs or intrinsic to processing can implement proper probabilistic computations. As previously stated, however, further analysis is required before we can definitively accept or reject this conjecture.

## Conclusion and future directions

This article has covered a lot of ideas related to Bayesian inference, generative models, and neural networks. The primary goal was to review the ideas necessary to establish the proposition that interactive neural network models and principled probabilistic models of cognition can be compatible with each other. I hope that this review fulfills this goal, and I also hope that it will be of broader use. The probabilistic and neural network concepts considered here are in broad use throughout the psychological, cognitive science, and cognitive neuroscience literatures, and their integration should help advance our understanding of probabilistic computation in perception and its implementation in neural systems.

For the future, there is exciting work to be done. To date the MIA model has been used primarily to establish the basic theoretical point that interactive computations in neural networks are completely consistent with principled Bayesian computations. The ability of the model (or a successor) to capture specific patterns in data, such as those captured by the original IA model of letter perception (McClelland and Rumelhart, 1981) and to capture the many findings in the literature that were not adequately addressed by the original model [e.g., the time-course of stimulus and context effects, as observed in Massaro and Klitzke (1979)] remains to be explored [initial steps in this direction were described in Khaitan and McClelland (2010)]. For that exploration, it will be necessary to develop, among other things, assumptions about exactly how the visual display conditions used in letter and word perception experiments affect activations of feature units and how this in turn affects the process of settling. Establishing more detailed links with the details of the underlying neurobiology will also be an important direction for the future.

I believe that incorporating learning and distributed representations will also be necessary to fully capture interactive processes in perception as they arise in naturalistic settings. We have seen in this article how an explicit generative model can be embedded in a perceptual system, so that it can sample from the generative model's posterior distribution. For this case, we have had the advantage of working in a domain—the domain of printed words and letters—where the relevant underlying units (the words and letters themselves) and contingent relations between them (letters depend on words, and features on letters)—can be identified, so that an explicit generative model (albeit oversimplified) can be advanced, and instantiated in a neural network. Real scenes that we are called upon to perceive are of course far more complex. There may be several objects in a display at the same time – so rather than a single underlying cause, there can be several. The underlying causes may be partially, but perhaps not completely, independent. The objects may take various poses and scales, be subject to various occluders and misleading lighting effects, etc. The objects themselves might not be fully characterized by mutually exclusive discrete identities, as words and letters are. To handle such cases, one can well imagine that no explicit generative model could ever do full justice to the actual entities or contingent probabilities involved.

A solution to this problem may involve using a network in which the units and connection weights are not pre-assigned, but learned. The network could still be viewed as instantiating a generative model, but without the prior stipulation of the correct set of units or connections. This is the approach taken in the deep belief networks introduced by Hinton and Salakhutdinov (2006). Incorporating these ideas into interactive models addressing the psychological and neural mechanisms of perception provides an exciting future challenge.

### Conflict of interest statement

The author declares that the research was conducted in the absence of any commercial or financial relationships that could be construed as a potential conflict of interest.

## Footnotes

^1^Often the word *data* is used instead of *evidence*. Some writers use evidence to refer to quite a different concept.

^2^By convention, we use Σ_*s*_ to refer to a sum of terms indexed by a subscript *s* and we use Π_*s*_ to refer to a product of terms indexed by *s* (in the equation here the subscript is *i*′ for consistency with later usage as explained below). A summation or product applies to all of the factors multiplied together following the summation symbol. Thus Σ_*i*_*a*_*i*_*b*_*i*_*c*_*i*_ is equivalent to Σ_*i*_(*a*_*i*_*b*_*i*_*c*_*i*_) and Σ_*i*_*a*_*i*_Π_*j*_*b*_*ij*_*c*_*ij*_ is equivalent to Σ_*i*_(*a*_*i*_Π_*j*_(*b*_*ij*_*c*_*ij*_)). Unless explicitly indicated with parentheses, summation does not extend across a plus or minus sign. Thus Σ_*i*_*a*_*i*_ + *b* is not the same as Σ_*i*_(*a*_*i*_ + *b*).

^3^It is worth noting that, if one tabulated counts of occurrences of letters in a randomly chosen book, the counts could be considered to be independent quantities. In this case, the total count of letters in the book would be the sum of the counts for all of the letters. If we were to be given this total count, we could infer the count for any given letter from the counts for the others letters and the total count. Either way, there are *N* + 1 quantities (each count and the sum) but only *N* independent quantities.

^4^Note that in general, ∑_*i*′ ≠ *T*_*p*(*F*_*ht*_|*l*_*i*′_)*p*(*l*_*i*′_) may not be equivalent to ∑_*i*′ ≠ *T*_*p*(*F*_*ht*_|*l*_*i*′_) ∑_*i*′ ≠ *T*_*p*(*l*_*i*′_).

^5^Strictly speaking, this formula is valid if the causes that could lead to missing evidence—e.g., no information about whether a particular feature is present or absent—are independent of the process that generates the feature values. This would be true if, for example, the probability that an occluder would partially restrict the visibility of an object were independent of the identity of an object.

^6^Those wanting to gain familiarity with these functions can obtain values by reading off of these functions, and check that the above relationships all hold up. For example log(2) + log(3) = log(2 · 3) = log(6) and log(8) − log(4) = log(8/4) = log(2), and not log(4).

^7^It may be useful to note that what determines how likely it is that the unit with the strongest net input is the most active unit is not the absolute magnitude of the noise but the ratio of the strength of the noise to the size of the difference in the net inputs to different units. Given this, the noise might remain constant, while the net inputs (and therefore differences among them) might build up gradually over time. In this way, as time progressed, we could go from chance performance to matching, and, if signals continue to grow stronger, to maximizing as a function of time spent processing.

^8^In principle, for each letter in each word we could have a full table of 26 times 4 entries, *p*(*l*_*j*_*p*__|*w*_*i*_), but for simplicity we will assume (as in Mirman et al., in press), that *p*(*l*_*j*_*p*__|*w*_*i*_) is the same, and is equal to a fixed parameter value *c*_*L*|*W*_ if *l*_*j*_ is the correct letter in position *p* of word *i* and that the remaining probability, 1 − *c*_*L*|*W*_, is split evenly among the remaining 25 letters. Thus, if *c*_*L*|*W*_ = 0.95, the value of *p*(*l*_*j*_*p*__|*w*_*i*_) for the incorrect letters will be 0.05/25 = 0.002. Using these numbers, with probability 0.95^4^ ≃ 0.81 all four letters generated from the chosen word will be the correct letters, but with probability 1 − 0.95^4^ ≃ 0.19 there will be one or more incorrect letters.

^9^Again for simplicity, we use a single parameter for correct features, *c*_*F*|*L*_; for incorrect features, the corresponding probability is 1 − *c*_*F*|*L*_.

^10^Note the distinction between {*v*_*dp*_}, the full set of indices of the active feature value units across all dimensions and all positions, and {*v*_*d*_*p*__}, the set of indices of the active feature values in position *p*.

^11^This law states that for all *a*, *b*, *c*, *d*: (*a* + *b*)(*c* + *d*) = *ac* + *ad* + *bc* + *bd*.

^12^It is worth noting that the simplification here dramatically speeds calculation. Instead of computing 26^4^ separate products of four quantities and then adding these all up, we compute the product of four sums of 26 quantities, producing a speed up of 17,000:1. It is also easier to implement the add-then-multiply simplification as a parallel computation in a neural network.

^13^In both the numerator and denominator of this equation and the next one, there is a line break before the product symbol ∏.

^14^In a sense the flow is still both bottom up and top-down, but there is no back-and-forth communication, which is the essence of interactive activation.

^15^It could be worth noting that we have the option of normalizing the above quantities for each word by dividing them all by their sum. This step will not make any difference, since ratios of these quantities will be used later in calculating posterior probabilities at the letter level.

^16^The importance of keeping these messages distinct becomes even more clear if the top-down signals need to be passed down more than one level, a possibility that arises in Pearl's general formulation.

^17^To the human eye, the features in position 1 seem consistent with the letter B as well as the letters F and H. However, in the Rumelhart and Siple font, B does not have a vertical on the left, so that letter is ruled out by the given features. Also, humans appear not to entertain the possibility of W in the third position, perhaps because the segments appear to terminate at the bottom, but again, the given features are equally consistent with W and X in the Rumelhart and Siple font.

^18^The word HEW is also consistent with one letter in each position, but it is very low in frequency and for the sake of the example we assume it is not known to the perceiver.

^19^Instead of goodness, Hinton and Sejnowski (1986), included a minus sign and called the quantity energy, following (Hopfield, 1982), but the equation is otherwise the same.

^20^It should be noted that some of the transition probabilities can be very small, and thus many of the transitions are highly unlikely. As we shall see below, we will not be relying on moving widely across the state space during processing of a single item.

^21^Being ergodic, as noted in footnote 18, is an in-principle matter, and some of the transition probabilities can be very small, but the starting state we start from—all units off except the clamped feature units—makes for easy access to all of the states that are plausible given the input.
